# Mammalian copper homeostasis: physiological roles and molecular mechanisms

**DOI:** 10.1152/physrev.00011.2024

**Published:** 2024-08-22

**Authors:** Svetlana Lutsenko, Shubhrajit Roy, Peter Tsvetkov

**Affiliations:** ^1^Department of Physiology, Johns Hopkins Medical Institutes, Baltimore, Maryland, United States; ^2^Department of Pathology, Cancer Center, Beth Israel Deaconess Medical Center, Boston, Massachusetts, United States

**Keywords:** ATP7A, ATP7B, brain, cuproptosis, SLC31A1

## Abstract

In the past decade, evidence for the numerous roles of copper (Cu) in mammalian physiology has grown exponentially. The discoveries of Cu involvement in cell signaling, autophagy, cell motility, differentiation, and regulated cell death (cuproptosis) have markedly extended the list of already known functions of Cu, such as a cofactor of essential metabolic enzymes, a protein structural component, and a regulator of protein trafficking. Novel and unexpected functions of Cu transporting proteins and enzymes have been identified, and new disorders of Cu homeostasis have been described. Significant progress has been made in the mechanistic studies of two classic disorders of Cu metabolism, Menkes disease and Wilson’s disease, which paved the way for novel approaches to their treatment. The discovery of cuproptosis and the role of Cu in cell metastatic growth have markedly increased interest in targeting Cu homeostatic pathways to treat cancer. In this review, we summarize the established concepts in the field of mammalian Cu physiology and discuss how new discoveries of the past decade expand and modify these concepts. The roles of Cu in brain metabolism and in cell functional speciation and a recently discovered regulated cell death have attracted significant attention and are highlighted in this review.

## Graphical abstract

**Figure:**
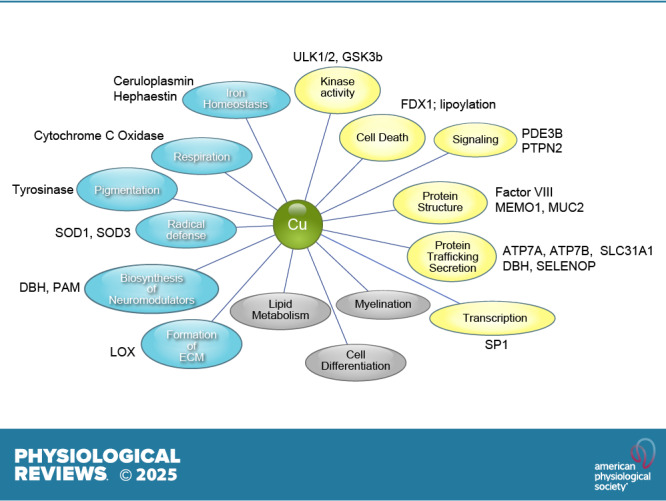

CLINICAL HIGHLIGHTS

- Copper homeostasis is essential for human physiology, and either copper deficiency or copper excess has detrimental effects on human health.
- Dietary copper deficiency is rare but can be caused by bariatric surgeries, parenteral nutrition, or ketogenic diet. The most common manifestations of systemic copper deficiency are anemia and neutropenia.
- Copper deficiency due to inborn mutations in the copper transporters ATP7A and SLC31A1 cause neurodevelopmental disorders: Menkes disease and fatal congenital copper transport defect, respectively. Copper histidinate and, on an emergency basis, copper elesclomol are being used for copper supplementation in newborns with these diseases.
- Wilson’s disease is caused by inactivating mutations in the copper transporter ATP7B. Patients with Wilson’s disease have a broad spectrum of pathologies, including liver disease and neurologic and psychiatric impairment. Clinical trials testing gene therapy for Wilson’s disease are underway.
- Cuproptosis, a recently discovered regulated cell death induced by copper ionophores, is being actively investigated as a new approach to treating cancer.
  Altered copper levels are reported in obesity, Parkinson’s disease, Alzheimer’s disease, and hematologic malignancies and can contribute to the pathogenesis of these disorders.

## 1. Cu IS A MULTIFACETED PLAYER IN CELL PHYSIOLOGY

Organisms in all phyla (from bacteria to humans) use copper (Cu) for numerous physiological processes. In this review, we focus on the role of Cu in mammalian cells while acknowledging the existence of vast literature, which illustrates the essential role of Cu in plants, fish, fungi, flies, worms, and many microorganisms. We will summarize only briefly topics that have been extensively reviewed in recent years and discuss in more detail new findings and emerging paradigms. It is not possible to cite all the work on mammalian copper biology, as more than 20,000 articles have been published in the past decade on this topic. We hope that this review of representative literature will serve as a useful primer to further reading on various aspects of Cu physiology, as this field is rapidly expanding and evolving.

### 1.1. Cu Has Unique Chemical Properties and Functionally Interacts with Other Metals

During evolution, Cu became an indispensable component of cell physiology owing to its unique chemical properties. Unlike most physiologically relevant metals, Cu accepts and donates electrons under physiological conditions and cycles between the two oxidation states, cupric Cu^+2^ and cuprous Cu^+1^. Important metabolic enzymes use this property of Cu in various reactions: to activate oxygen and make it biochemically available, to transfer electrons between molecules, to detoxify radicals, and to stabilize substrates within their binding sites (1–3). Iron (Fe) is another redox active metal, and it is more abundant than Cu. However, in cells iron and Cu are not interchangeable under most circumstances. They have preferred cellular sites of action, distinct chemical characteristics including different sets of major oxidation states (for iron these are Fe^2+^ and Fe^3+^), and different coordination environments in their binding sites (4). In fact, some enzymes, such as cytochrome *c* oxidase, require both metals to facilitate catalysis (5, 6). Despite distinct properties, the homeostasis of Fe and Cu are intertwined (7). The export of iron from cells depends on the activity of Cu-dependent ferroxidases ceruloplasmin and/or hephaestin (8–10). Consequently, Cu deficiency can cause iron misbalance in tissues and lead to anemias, among other symptoms (11–13). Cu excess is also detrimental to iron homeostasis, because Cu may disrupt the formation of iron-sulfur clusters in enzymes and inhibit their activities (14, 15).

Homeostasis of another biological metal, zinc (Zn), is also interconnected with Cu (16–20). Unlike Fe, Zn uses the same amino acid residues as Cu (Cys and His) for binding to proteins. Excess Cu can displace Zn from its binding sites in proteins and alter their structure and activity (16). Reciprocally, excessive use of Zn (for example in dietary supplements) can upregulate metal-chelating proteins metallothioneins (MTs). MTs bind both Zn and Cu, but the affinity for Cu is significantly higher (21). Therefore, Zn-induced upregulation of MTs can drastically decrease Cu availability (18–20). This latter property found clinical applications in the treatment of Cu overload in Wilson’s disease (WD), with a variable degree of success (22, 23). Specific Cu-carrying proteins, called Cu chaperones, have evolved to deliver and insert Cu into enzymes that have binding sites for more than one metal (Zn and Cu or Fe and Cu, see more below).

Recent data suggest that in addition to Cu and Zn binding to some of the same proteins, such as MT1 and MT2 (24, 25), the cell’s response to drug treatments can involve changes in both metals. For example, clioquinol increases the cellular content of both Zn and Cu and affects the behavior of Cu-binding proteins (26). Elucidation of functional interactions between Cu, Fe, and Zn will help to understand their normal physiology and contributions to diseases [cancer, multiple sclerosis, and Alzheimer’s disease (AD)], in which the misbalance of all these metals was reported (27–31). Currently, studies of metal dys-homeostasis in human diseases often focus on individual metals rather than the entire metallome.

### 1.2. Biological Roles of Cuproenzymes Are Expanding

The best-known and arguably the most important function of Cu is to serve as a cofactor to various metabolic enzymes. Some Cu-dependent enzymes are ubiquitous, i.e., found in most cell types, whereas others are expressed preferentially in highly specialized cells (TABLE 1). There is a large disparity in the amount of biochemical, cellular, and genetic information available for different Cu-dependent proteins in specific cell types. For example, dopamine-β-hydroxylase (DBH) has been extensively characterized in vitro and in vivo (32–37), whereas information on a structurally similar DBH-like monooxygenase X (MOXD1) is very limited (38). The recent discovery of the MOXD1 role in the proliferation of tumor cells (39) and early pulmonary fibrosis (40) promises to bring more attention to the function and regulation of this intriguing enzyme.

**Table 1.** Function and cell-specific expression of Cu-dependent enzymes

Enzyme	Function	Expression
Cytochrome *c* oxidase (MT-CO2)	Electron transfer and respiration	Ubiquitous
Cu/Zn-dependent superoxide 1 (SOD1)	Detoxification of intracellular superoxide	Ubiquitous
Superoxide dismutase 3 (SOD3)	Detoxification of extracellular superoxide	Lung and blood vessels
Cu-dependent amino-oxidase 1 (diamine oxidase) (AOC1)	Oxidation and degradation of polyamines: putrescine, histamine, spermine, and spermidine	Gastrointestinal, kidney, reproductive tissues, and bone marrow
Cu-dependent amino-oxidase 2 (AOC2)	Monoamine oxidase activity with substrate specificity for 2-phenylethylamine and tryptamine	Broadly expressed and enriched in the liver, testis, and adipose tissue
Cu-dependent amino-oxidase 3 (AOC3)	Monoamine oxidation, lymphocyte extravasation, and uptake of metabolic fuels in adipocytes	Adipocytes, vasculature, lung, bladder, and breast
Dopamine-β-hydroxylase (DBH)	Conversion of dopamine to norepinephrine	Locus coeruleus and adrenal gland
DBH-like mono-oxygenase (MOXD1)	Hydroxylation of hydrophobic substrates in the endoplasmic reticulum (proposed)	Salivary gland, ovary, brain, nasal cavity, pituitary, and heart
Peptidyl-glycine alpha-amidating monooxygenase (PAM)	Amidation of signaling peptides	Heart, brain, lungs, gastrointestinal, kidney, glands, and bone marrow
Lysyl oxidase (LOX)	Deamination of lysine and hydroxylysine residues of collagens and elastin	Connective tissue, respiratory system, muscle, adipose tissue, liver, and gallbladder
Lysyl oxidase homolog 1 (LOXL1)	Deamination of lysine and hydroxylysine residues of collagens and elastin	Heart, urinary bladder, and placenta
Lysyl oxidase homolog 2 (LOXL2)	Deamination of lysine and hydroxylysine residues of collagens and elastin	Many tissues, reproductive tissues, placenta, uterus, and prostate
Lysyl oxidase homolog 3 (LOXL3)	Deamination of lysine and hydroxylysine residues of collagens and elastin	Bone marrow, placenta, spleen, and smooth muscle
Lysyl oxidase homolog 4 (LOXL4)	Deamination of lysine and hydroxylysine residues of collagens and elastin	Many tissues, especially salivary gland, gastrointestinal, muscle, and cervix
Tyrosinase (TYR)	Pigmentation and melanin synthesis	Skin, eye, and hair
Ceruloplasmin (CP)	Ferroxidase: facilitates iron efflux from the cell	Liver and macrophages
Hephaestin (HEPH)	Ferroxidase: facilitates iron efflux from the cell	Intestine
Mediator of cell motility (MEMO1)	Cu(I) binding, iron binding, and role in iron homeostasis	Ubiquitous, testis, skeletal muscle, liver, and bone marrow
Serine/threonine-protein kinase (ULK1 and ULK2)	Regulation of autophagy	ULK1: ubiqiotous/muscle; ULK2: testis
Selenium binding protein P (SELENBP1)	Methanethiol oxidase: converts methanethiol to H_2_O_2_, H_2_S, and formaldehyde	Colon

Rich new biology can be uncovered by studying the well-known cuproenzymes in the previously uncharacterized cell types or in different organisms. This point can be illustrated by a stream of recent discoveries related to peptidyl alpha-amidating monooxygenase (PAM). This highly conserved and extensively studied enzyme uses two Cu atoms and ascorbate to catalyze the hydroxylation and amidation of the COOH-terminal Gly residue in biologically active peptides. Amidation stabilizes the peptides and alters their structure to enhance signaling function (41–43). PAM activity is critical for organism survival, as evident from the embryonic lethality of PAM knockout mice. *Pam^+/-^* heterozygous animals survive but exhibit a spectrum of behavioral phenotypes accompanied by enhanced excitability and deficient synaptic plasticity in the amygdala (44). Recent studies identified new fascinating roles for PAM: a pH sensor and regulator of V-type ATPase assembly within the endocytic pathway of peptidergic cells (45), a regulator of the cytoskeleton (46), a required factor in ciliogenesis (47, 48), and a contributor to the biogenesis of atrial granules (49). Some of these functions depend on PAM enzymatic activity (and hence Cu availability), whereas others do not. Thus Cu misbalance is likely to have nonuniform consequences on PAM-dependent processes, which is important to consider, especially when studying Cu deficiencies.

In many cases, even when Cu-containing enzymes have a well-established role in an organism’s physiology, their other Cu-related properties, such as the delivery mechanism of their Cu-cofactor, Cu dependence of expression, and/or localization in a cell, and the contribution of these enzymes to overall cell Cu homeostasis remain mostly unknown. In studies of transcriptomes and proteomes, correlations are rarely made between the expression of Cu-dependent enzymes and proteins responsible for supplying their Cu cofactor or regulating their intracellular targeting, even though both processes are essential for the function of cuproenzymes. Uncovering these correlations is bound to increase the information content of many omics studies.

Recent work illustrates that exploring the metal-dependent regulation of Cu-containing enzymes may lead to unexpected and interesting discoveries. For example, vascular adhesion protein VAP-1, also known as Cu-dependent amino oxidase 3 (AOC3), has a well-established role in leukocyte adhesion during inflammation (50). VAP-1/AOC3 is also a Cu-dependent enzyme, which is abundantly expressed in adipose tissue and vasculature, where it catalyzes the conversion of small monoamines to aldehydes (51). This function is required for the monoamine-dependent increase in cellular glucose uptake (52). Studies of AOC3 in the context of Cu deficiency found that the activity of AOC3 is needed to balance the uptake of glucose and lipids in adipocytes (53). Pharmacologic or genetic decrease in the delivery of Cu cofactor to AOC3 causes an increased fat accumulation and adipocyte hypertrophy (53). In vivo, mice expressing inactive AOC3 have metabolic disturbances and dyslipidemia (54). AOC3 is a membrane-bound protein but can also be produced in a soluble form and found in serum; both soluble and membrane-bound AOC3 modulate glucose and lipid uptake (53). These findings raise an interesting possibility that a soluble AOC3, which is elevated in the serum of patients with obesity, steatosis, and cancer (50, 54–56), may regulate the metabolic status of tissues in addition to its well-established role in inflammation.

### 1.3. Ubiquitous Cu-Dependent Enzymes and Their Functions

Most cells use Cu for mitochondrial respiration, which is a ubiquitous and essential process. Cu-containing cytochrome *c* oxidate (MT-CO2 or complex IV) is the major component of the respiratory chain. MT-CO2 is located in the inner membrane of mitochondria and has two Cu-containing centers. A binuclear CuA center transfers electrons from cytochrome *c* to the Fe/heme-Cu(B) center of cytochrome *c* oxidase, where electrons are used to reduce molecular oxygen (5, 6). A specialized set of soluble and membrane-bound proteins (chaperones) work together to insert Cu into the CuA center (for detailed review, see Ref. 57). Loss of Cu incorporation into MT-CO2 and the associated loss of its activity are detrimental to mitochondria function and cell survival (58–61). Rare exceptions are erythrocytes, which lack mitochondria and rely on glycolysis for their energy needs. Interestingly, although mature erythrocytes are less dependent on Cu, in their precursor state, these cells require robust Cu homeostasis for functional maturation and hemoglobin biosynthesis (62).

Cu is a required cofactor of another ubiquitous and abundant enzyme: cytosolic Cu, Zn-dependent superoxide dismutase 1 (SOD1). SOD1 converts highly reactive superoxide, generated during an electron leak from the mitochondria respiratory chain, to less harmful peroxide and water (63). SOD1 is a dimer of two identical monomers, each of which has one Cu and one Zn cofactor. Zn plays an important structural role, whereas Cu is used for catalysis. Small amounts of SOD1 are also found in the inner membrane space of mitochondria (64). The detoxification of oxygen radicals in the mitochondria matrix is mediated by the Cu-independent, Mn/Zn-superoxide dismutase SOD2 (65).

Missense mutations in SOD1 are identified as the second most common cause of amyotrophic lateral sclerosis (ALS; Ref. 66), a fatal neurodegenerative disorder, associated with progressive degeneration and eventual death of motor neurons (67). Extensive studies of SOD1’s role in ALS yielded a plethora of possible mechanisms, involving SOD1 misfolding and aggregation in motor neurons, mitochondria malfunction, changes in Ca^2+^ signaling, and others; for a detailed review, see Ref. 68. Although the importance of SOD1 inactivation in ALS pathogenesis was initially questioned, recent studies of mice with genetic deletion of SOD1 found increased cell sensitivity to oxidative stress, myofiber atrophy, and degeneration of neuromuscular junctions associated with the loss of SOD1 function (69).

Lysyl oxidase (LOX) and four LOX-like proteins (LOXL1–4) are not truly ubiquitous, as some tissues produce them at higher levels than others, but they are broadly expressed and play an important role in the maintenance of extracellular cell matrix. Lysyl oxidases deaminate (oxidize) primary amines to aldehydes and thus enable protein cross linking (70). Oxidation of lysine residues, which leads to cross linking within collagen and elastin, is the best-known function of LOXs. Recent studies revealed additional roles of LOX enzymes in the oxidation of extracellular and intracellular targets, such as histones, the transcription factors TAF10 and SNAIL1, and others, significantly expanding the involvement of these enzymes (and therefore Cu requirements) in cell physiology (for review of LOX targets, see Ref. 71).

For their enzymatic activity, LOX proteins utilize a unique protein-based cofactor, lysine tyrosylquinone (LTQ). LTQ is generated via a Cu^2+^− and O_2_-dependent self-catalyzed modification of a conserved tyrosine residue (72–75). Thus, in LOX enzymes, Cu has a dual role: it facilitates the formation of the LTQ cofactor and then together with LTQ participates in the oxidation of protein substrates (76, 77). A Cu deficit is especially detrimental to LOX activity (78, 79). The malformation and fragility of connective tissues and blood vessels in Cu deficiency are well documented (80). Zn can prevent the formation of the LTQ cofactor, at least in vitro (77), further illustrating functional relationships between these two metals.

### 1.4. Specialized Cu-Dependent Enzymes Are Key Contributors to Cells’ Functional Identity

**FIGURE 1.**
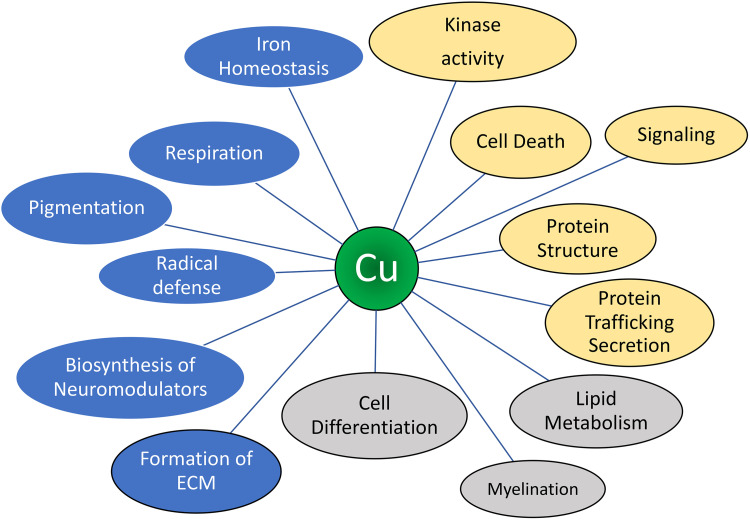
The multifaceted role of copper (Cu) in cell physiology. The cartoon illustrates (in blue) the role of Cu as an enzyme cofactor and corresponding physiological processes. In yellow: the role of Cu as a regulator of protein structure and function, which is mediated through direct binding to specific targets. In gray: the Cu-dependent processes for which importance of Cu is firmly established, but the mechanisms of Cu action are not yet fully understood. DBH, dopamine-β-hydroxylase; LOX, lysyl oxidase; MEMO1, mediator of cell motility 1; MUC2, mucin 2; PAM, peptidyl alpha-amidating monooxygenase; PDE3, phosphodiesterase 3.

Many Cu-dependent enzymes are enriched in specific cell types, which use these enzymes for their characteristic physiological functions (see Ref. 81 and FIGURE 1).

Noradrenergic neurons in the locus coeruleus and chromaffin cells of the adrenal gland are major sources of the neurotransmitter norepinephrine. These cells abundantly express dopamine-β-hydroxylase (DBH), also known as dopamine-β-monooxygenase. DBH uses two uncoupled Cu atoms to hydroxylate dopamine, a step necessary for the conversion of dopamine to norepinephrine (noradrenaline), an important hormone and neurotransmitter (41). Mice with a genetically inactivated DBH or humans with Cu deficit (Menkes disease patients) show catecholamine misbalance and an increased susceptibility to seizure-inducing stimuli (82, 83). Recent single-cell sequencing studies uncovered additional, unexpected, populations of cells expressing DBH. Among them are the subset of cardiomyocytes involved in the initiation and coordination of cardiac excitation and contraction (84) and the subset of hepatic stellate cells with a gene signature of antigen-presenting cells (85). Further studies of DBH function in these cell types including the Cu-dependent activation and regulation of DBH promise to expand our understanding of catecholamine physiology.

Ceruloplasmin (CP) is another example of a Cu-dependent enzyme with an expanding range of host cells as well as functions. CP plays an important role in iron homeostasis and inflammatory response (86). It has six Cu atoms arranged into three sites, and it couples the reduction of molecular oxygen to the oxidation of ferrous iron Fe^2+^ into a ferric Fe^3+^ form (87). The Fe^+2^ oxidation is necessary for iron efflux and transport between tissues, because the serum iron carrier transferrin only binds Fe^3+^. Hepatocytes, macrophages, and lactating mammary glands are major sites of CP production (88–90). In addition, recent studies found that glial cells express CP and that the CP expression in oligodendrocytes contributes to their functional maturation and myelin production (91). These findings not only expand the spectrum of cells with high CP abundance but may also explain the negative effects of Cu deficit on myelination (92, 93).

Cells of the skin, eye (retina), and hair express the Cu-dependent enzyme tyrosinase (TYR) and two related proteins, TRP1 and TRP2. Tyrosinase uses two Cu atoms to oxidize tyrosine (and other phenols) to quinone, a precursor of pigment melanin (94, 95), which protects cells against harmful consequences of UV light. Inactivation of tyrosinase is associated with oculocutaneous albinism 1 in humans (96, 97). In animals, the temperature-sensitive mutations, which cause tyrosinase misfolding and/or loss of its Cu cofactor, produce a characteristic Siamese coloring (98–100). In colder areas (ears, face, tail, and paws), the enzyme is folded and functional, resulting in pigment production and dark coloring, whereas in the rest of the body, it is less active yielding light pigmentation. Curiously, despite being generated through gene triplication and being highly homologous to tyrosinase, TRP2 is a Zn-dependent enzyme and is involved in the regulation of pigment abundance rather than melanin synthesis per se (101). The nature of the TRP1 cofactor is being debated (101). TRP1, similarly to a classic Cu-dependent tyrosinase, is important for pigmentation; however, unlike tyrosinase, it depends on Zn transporters for its expression and activity (102).

Superoxide dismutase 3 (SOD3) is another example of a Cu-dependent enzyme tightly linked to the major functions of cells, in which it is expressed. SOD3 is abundant in tissues exposed to high levels of oxygen, such as lungs and blood vessels. SOD3 is structurally and functionally similar to SOD1 (above) but performs its function (detoxification of oxygen radicals) in the extracellular space. Inactivation of SOD3 is associated with increased vascular inflammation and pulmonary hypertension (103) as well as delayed neovascularization and impaired cell signaling (104). SOD3 has only one Cu cofactor (no Zn) and can be either anchored to the plasma membrane or secreted (63). In agreement with its role in protecting vasculature, the virus-delivered soluble SOD3 has been shown to have therapeutic potential in preventing chemotherapy-induced oxidative stress (105). The role of secreted SOD3 is not yet understood.

For cell-specific Cu-dependent enzymes to contribute to characteristic functions of their host cells, their biosynthesis, including the insertion of Cu cofactor, has to be a part of a well-coordinated cell differentiation program. Recent data provide direct support for this hypothesis: specific changes in the intracellular Cu distribution were shown to accompany the production of Cu-dependent enzymes during cell differentiation (53, 106–108). Another prediction is that the pathologies involving the inactivation of Cu-dependent enzymes may arise not only from mutations in the enzyme per se but also from the defects in the delivery, incorporation, and retention of Cu (109). For example, mice lacking fibulin-4 have long been known to have abnormal elastic fibers and die from emphysema, artery hemorrhages, and ruptures (110). These symptoms are very similar to those observed in *Lox*-/- mice (111). Recently, it was discovered that fibulin-4 facilitates the formation of LTQ cofactor in LOX (112), which is also a Cu-dependent process (75). Therefore, fibulin appears to serve as a molecular “coordinator” between the Cu-delivery system (ATP7A, see below) and LOX.

Movement disorders represent another example of phenotypic similarities associated with the disruption of Cu-dependent and Cu-independent pathways. Parkinson’s disease (PD) can be caused by various factors (both genetic and nongenetic) resulting in a loss of substantia nigra neurons and motor disfunction (for recent comprehensive review, see Ref. 113). Parkinsonism (tremors, rigidity, and slow movements) is a common feature of Wilson’s disease, a disorder of Cu misbalance (114, 115). The precise mechanistic basis of the overlapping phenotypes between Parkinson’s disease and Wilson’s disease remains to be established. However, it is notable that catecholamine balance is altered in both disorders (116, 117) and that in either disease, substantia nigra is among the most affected regions of the brain.

### 1.5. Nonenzymatic Functions of Copper

In addition to serving as an enzyme cofactor, Cu has structural, regulatory, and signaling functions.

#### 1.5.1. Structural and allosteric roles of Cu.

The components of a coagulation cascade, factor VIII, and mucin MUC2 are extracellular proteins that bind Cu, possibly to stabilize their structure and/or to prevent futile Cu^+2^/Cu^+1^ cycling at the cell surface (118, 119). A similar function, stabilization of Cu^+1^, was proposed for the intracellular protein mediator of ERBB2-driven cell motility 1 (MEMO1; Ref. 120). More recent studies revealed significant genetic interactions of MEMO1 with proteins involved in iron metabolism (121). In vitro, in the presence of glutathione, MEMO1 binds either Fe^2+^ or Cu^+1^ with comparable affinities using the same coordinating residues (121). This result suggests that iron (which is significantly more abundant) is a primary metal cofactor of MEMO1 but also could be a target of Cu overload. Given growing evidence for the role of Cu in cell signaling and motility, additional Cu-dependent functions of MUC2 and MEMO1 are likely to be discovered. Transcription factor SP1 contributes to the regulation of the Cu transporter expression (122, 123) and was shown to tightly bind copper (124). Cu also binds to the autophagic ULK1 kinase and allosterically stimulates its activity (125), whereas GSK3B kinase is inhibited by Cu elevation in cells (126). Other Cu-regulated kinases were proposed to exist (127, 128); however, for most of them, the biochemical mechanism of Cu-dependent regulation is not yet fully understood (for detailed review, see Ref. 129).

#### 1.5.2. Cu as a regulator of protein trafficking.

The binding of Cu^+1^ to regulatory domains of the Cu transporters ATP7A and ATP7B causes conformational changes within these domains, which in turn lead to kinase-mediated phosphorylation, altered interdomain interactions, and changes in the intracellular localization of ATP7A and ATP7B (130–134). Cu depletion reverses these effects. Similarly, Cu binding to the Cu-transporter SLC31A1 (also known as CTR1) triggers its internalization, and Cu depletion returns the transporter to the plasma membrane (see also sect. 2.3). In addition to regulating the intracellular localization of Cu transporters, Cu modulates protein secretion. Cu elevation inhibits the synthesis and secretion of selenoprotein P (135, 136). In adipocytes, the negative effects of genetically induced Cu overload on selenoprotein abundance can be reversed by lipoic acid, which increases cellular selenium (Se) content, or by Cu chelation (137). Cu also regulates the constitutive secretion of DBH. In a cellular model of noradrenergic neurons (SHSY-5Y cells), the inactivation of ATP7A or ATP7B has the opposite effect on DBH secretion (116). Overexpression of ATP7B, which sequesters Cu from the cytosol, or Cu depletion using an extracellular Cu chelator bathocuproine disulfonate and an intracellular Cu chelator, tetrathiomolybdate, causes retention of DBH in SHSY-5Y cells suggesting Cu requirement for DBH export. Consistent with this model, the downregulation of ATP7B, which increases Cu levels in the cytosol, stimulates DBH secretion (116).

#### 1.5.3. Cu involvement in cell-to-cell communications and signaling.

Experiments in rat hippocampal neurons using radioactive Cu^67^ demonstrated a calcium-dependent release of Cu^67^ in response to potassium-induced depolarization (138). Similarly, activation of the NMDA receptor results in the release of Cu into a synaptic cleft (139). These results led to the suggestion that Cu modulates neuronal activity (140). The molecular form in which Cu is released from neurons [a free Cu ion, Cu bound to small molecule(s)] or Cu bound to proteins like DBH (116, 141) is still unknown. Determining the molecular nature of the Cu-based signals is needed to understand the mechanism of Cu-dependent cell-to-cell communication. Currently, the physiologic relevance of effects elicited by the exogenously added Cu on various channels (142) is uncertain, because even a modest elevation of free Cu greatly exceeds free Cu levels found in the interstitial fluid.

Intracellular Cu can modulate receptor-mediated signaling by altering the activity of Cu-dependent effectors. For example, Cu inhibits cAMP-degrading phosphodiesterase PDE3, which acts downstream of β-adrenergic receptor and decreases lipolysis (143). Changes in Cu levels also regulate the suprachiasmatic circadian clock and a day-night rhythm in the expression of the Cu transporter ATP7A (144). Expression of another Cu transporter, a truncated isoform of ATP7B, is controlled by diurnal rhythms in the pineal gland and retina, indicative of rhythmic changes in Cu homeostasis (145). In the pineal gland, both a- and b-pinealocytes express ATP7B, which is among the most induced genes during the night or in response to isoproterenol (146). Repeated injection of mice with Cu during either “light on” or “lights off” time produces more toxic effects at the latter (lights off) schedule further illustrating a link between Cu metabolism and circadian rhythms (147).

#### 1.5.4. Short-term and long-term effects of Cu misbalance.

In addition to regulatory events that take place within minutes and require only small changes in intracellular Cu concentration, significant cell responses are induced by prolonged changes in cellular Cu levels. Dietary Cu deficiency is rare, but an increasing number of individuals have been reported by physicians to have Cu deficiency after bariatric surgery and other surgical interventions causing malabsorption or due to unknown causes (148–151). The common manifestations of Cu deficiency, anemia, neutropenia, ataxia, and myopathies, are well documented. However, why Cu deficit is particularly harmful to the neutrophil count or to the health of motor neurons is less clear, although the negative effect on mitochondria cytochrome *c* oxidase can be a contributing factor (152–155). The effects of high Cu are multifaceted and have been explored in more detail. Cu elevation inhibits mitochondria function (156) and alters the nuclear-cytosolic shuttling of RNA-binding proteins (157) and b-catenin (158). Elevated Cu also induces autophagy (159), impacts the length of the 3′-end of mRNAs during cell differentiation (160), and inhibits nuclear receptor function (161–163). High Cu triggers epigenetic reprogramming that impacts DNA methylation and immune cell response (164). Further studies will determine whether any of these adaptations to chronically elevated Cu may also occur at a time scale of regulatory events.

Cu appears to directly interact with the components of the cytoskeleton and alter cell shape and motility (165–167). In vitro, Cu^2+^ induces fragmentation and polymerization of actin (168). However, in cells, copper exists primarily in the Cu^+1^ state, and therefore, it is unclear whether the observed Cu^2+^ effects are physiological or pathophysiological (169). Copper was also shown to regulate the activity of splicesosome (170), to increase the sensitivity of tRNA-splicing ligase to oxidative inactivation by H_2_O_2_ (171), and to induce changes in the splicing pattern of RNA isoforms (157). The biochemical details for these Cu effects remain to be investigated. Mitochondria is particularly sensitive to either Cu deficiency or Cu overload. Treatment of cells with Cu chelators decreases the ratio of mitochondria-to-glycolysis ATP production, destabilizes complex IV (cytochrome *c* oxidase), and decreases cells’ invasiveness and ability to migrate (152). Further studies have found that Cu depletion mediates these effects via the activation of AMPK and reduced activity of mammalian target of rapamycin (152). While significant Cu deficit decreases cytochrome *c* oxidase activity, excess Cu in mitochondria can cause loss of iron-sulfur clusters (14), protein thiol oxidation, membrane protein cross linking, and, in extreme cases, mitochondria destruction (172). Recent studies revealed a role for copper in mitochondria flickering, which is a safeguard mechanism to prevent excessive mitochondria fusion. Flickering reflects short pulses of mitochondria depolarization, which activate Oma1 protease located in the inner membrane of mitochondria. Oma1 degrades a mitochondrial fusion protein Opa1 and limits mitochondrial fusion (173). Reciprocally, in erythroid cells, a Cu deficit upregulates Opa1 and increases mitochondria fusion (62, 174).

## 2. COPPER DISTRIBUTION BETWEEN TISSUES

### 2.1. Cu Levels in Tissues Are Not Uniform

**FIGURE 2.**
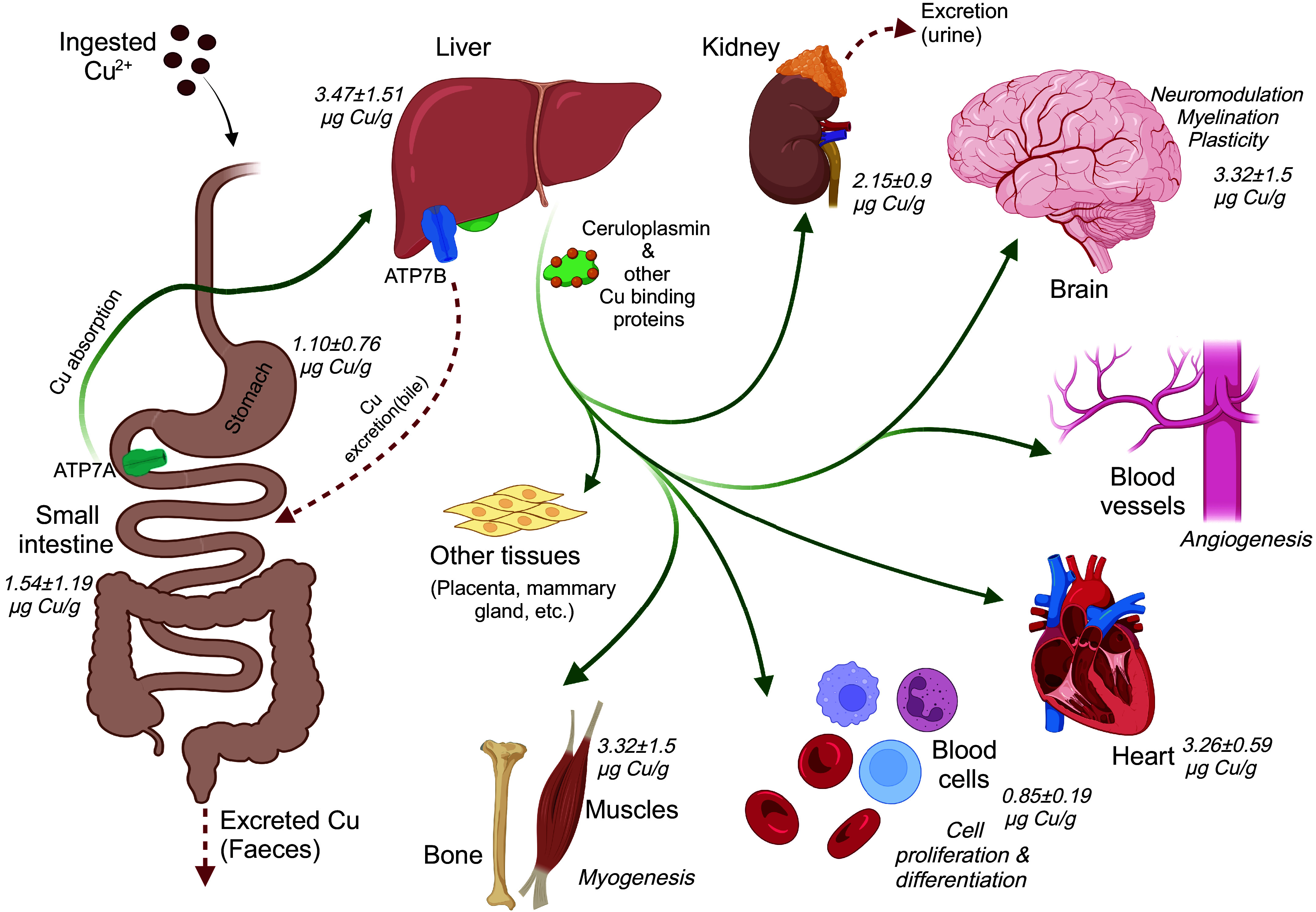
Copper (Cu) is absorbed from the dietary sources by the small intestine and transported to tissues, with the liver being the major recipient of incoming Cu. The liver has the highest Cu concentration (3.47 µg/g wet tissue). In the liver, Cu is incorporated into ceruloplasmin, which is then secreted into the bloodstream. Excess Cu is exported into bile and removed with feces. The brain also has high Cu content (3.32 µg/g wet tissue). In other tissues, Cu plays a crucial role in angiogenesis, cardiac development, hematopoiesis, inflammatory response, and myogenesis. Image created with BioRender.com, with permission.

Copper enters a human or animal’s body via the stomach, duodenum, and the proximal segment of the jejunum, with the duodenum being the major site of Cu uptake (175). After exiting the gastrointestinal tract, Cu is quickly, within 10 min, distributed to tissues; over 50% of total Cu is taken up by the liver (176). At a steady state, the liver, brain, heart, and kidneys, in decreasing order, have the highest Cu content per gram of tissue (FIGURE 2). Cu levels are intermediate in the lung, intestine, and spleen, whereas endocrine glands, muscle, and bone have the lowest Cu concentrations (177). Although Cu concentration in the muscle and bones is low, because of their mass, these tissues together contain ∼50% of total body Cu. The liver uses Cu to synthesize and secrete Cu-containing ceruloplasmin (CP), causing a second wave of Cu elevation in serum within 2 hours post-Cu ingestion (178) and the maximum level within 48 hours (179). Recent PET/CT studies in human subjects explored the initial (10–90 min after ingestion) Cu distribution between tissues. In addition to tissues with the expected high Cu entry, such as the liver, an unexpectedly high uptake of Cu was seen in subcutaneous fat (7–9% of total; Ref. 176). This observation is especially intriguing given recent reports on the role of Cu in the formation of chylomicrons (the dietary fat carriers; Ref. 180) and in enhancing lipolysis and fatty acid oxidation in adipose tissue (181). Whether chylomicrons carry Cu and lipoproteins can regulate Cu uptake by tissues is presently unknown.

### 2.2. Serum Copper-Containing Components and Their Role in Cu Transfer to Tissues

#### 2.2.1. High-molecular-weight components: ceruloplasmin and albumin.

The form in which Cu is released from the intestine into the blood and then presented to tissues remains a matter of uncertainty. Owning to ceruloplasmin (CP) high abundance, the Cu cofactors bound to CP constitute the vast majority of total serum Cu. Early work suggested that CP could be involved in the delivery of Cu to tissues; however, later studies of CP-/- mice ruled out this hypothesis, as no defects of Cu homeostasis were observed in these mice (182). Another abundant serum protein, albumin (Alb), binds Cu^2+^ in vitro via the NH_2_-terminal ATCUN (H_2_N–X-X-His) motif; this Cu can be reduced in the presence of ascorbate to Cu^+1^ and be potentially a source of Cu for tissues (183). It is unclear, however, whether in vivo albumin binds “free” Cu or carries complexes of Cu with some metabolites or other small molecules (184). The large disparity between the concentrations of albumin and Cu in serum (35–50 mg/mL or 0.5–0.75 mM and 0.7–1.4 μg/mL or 11–20 mM, respectively) and the ability of albumin to inhibit Cu uptake in vitro (185) create uncertainty about the effectiveness of albumin as a Cu-delivery agent. The minor role of albumin in the distribution of Cu to tissues is also evident from the fact that *Alb*-/- mice are healthy in contrast to severely compromised mice with Cu deficiencies caused by dietary restrictions or genetic mutations in Cu transporters (186).

#### 2.2.2. Non-ceruloplasmin copper and a small copper carrier.

The term “nonceruloplasmin copper” (NCC) is traditionally used to describe all serum Cu components, except CP (176, 187, 188). Studies using chromatography coupled to inductively coupled plasma mass spectrometry (ICP-MS) determined that in healthy individuals CP accounts for 85% of Cu, albumin for 6%, and low-molecular-weight components account for ∼9% of total Cu in serum (189). Cu bound to small molecule(s), dubbed the small copper carrier (SCC), was also found in the urine (190, 191). Whether the low-molecular-weight components of NCC and SCC are the same molecule(s) is still unclear, although both are elevated in patients with liver copper overload (176, 192). The in vitro measurements of Cu^67^ uptake demonstrated the ability of a chromatographically enriched SCC to complete with free Cu for the uptake by cells at physiologically relevant concentrations of 1–2 mM (190, 191). These findings suggest that SCC may act as a Cu source for tissues, while its excess is filtered into the urine, but more research is needed. In Wilson’s disease patients, both the relative abundance of NCC compared to CP and the amount of SCC in the urine increased, and a similar observation was made for Parkinson’s disease patients (193, 194). In cancers, NCC can be elevated (187) even when total copper is not (195, 196). Whether the elevation of NCC or only its low-molecular-weight component (nonbuffered by albumin) results in the elevation of copper uptake by tissues remains to be determined.

The Cu content of serum and tissues can be affected by diet, environmental exposure, gender, age, and normal genetic variability in proteins involved in Cu absorption, utilization, or storage. Genetic linkage analysis identified two loci on chromosome 1, which contain genes that may modify concentrations of Cu in the blood (197). One region of association included the genes CCDC27, LOC388588, and LRRC47. The second locus showing a highly significant association with the erythrocyte Cu levels (*P* = 2.6 × 10^−20^) contains the gene selenium binding protein P (SELENBP1). Recently, SELENBP1 was shown to be a Cu-dependent enzyme abundantly expressed in the gastrointestinal tract, lungs, and bone marrow (198). SELENBP1 catalyzes the conversion of methanethiol to hydrogen peroxide, hydrogen sulfide, and formaldehyde (84). How changes in SELENBP1 activity influence the blood Cu content is a question for further studies.

### 2.3. Tissues Communicate Their Cu Status

Another intriguing finding that awaits further investigation is the ability of tissues to communicate their need for Cu through so far unidentified signaling molecule(s) released into the blood. Genetically induced Cu deficiency in a heart causes upregulation of Cu efflux from the liver into the blood, presumably to provide Cu supplementation to Cu-deficient cardiac tissue (199). Systemic Cu deficiency (induced by the diet) decreases Cu efflux from the peripheral tissues (to preserve tissue Cu content) but upregulates Cu efflux from the intestine into the bloodstream to increase Cu supply to the periphery (200). This is achieved by the opposite changes in the abundance of Cu transporter ATP7A in the intestine and at the periphery (200). During microbial infection, kidneys release Cu into the bloodstream to be utilized by the liver for an increased production of CP (201). The molecular mechanisms behind these signaling events are poorly understood, and recently developed genetic tools for studies of tissue-specific secretomes (202, 203) may help to identify signaling molecules that communicate the tissue Cu status.

## 3. COPPER TRANSPORTERS AND CHAPERONES ARE MAJOR REGULATORS OF Cu HOMEOSTASIS

### 3.1. Copper Transport into Cells

Copper uptake from the bloodstream into cells is mediated predominantly by the high-affinity copper transporter SLC31A1, traditionally called CTR1. The importance of CTR1 in mammalian physiology is evident from the embryonic lethality of mice with a genetically deleted CTR1 (204, 205) and severe neurological impairment of human patients with mutations in this transporter (206, 207). Furthermore, the 3′-untranslated region of CTR1 mRNA contains genetic polymorphisms that correlate with the survival time of cancer patients and their sensitivity to a platinum-based therapy (208).

#### 3.1.1. CTR1 structure and mechanism.

**FIGURE 3.**
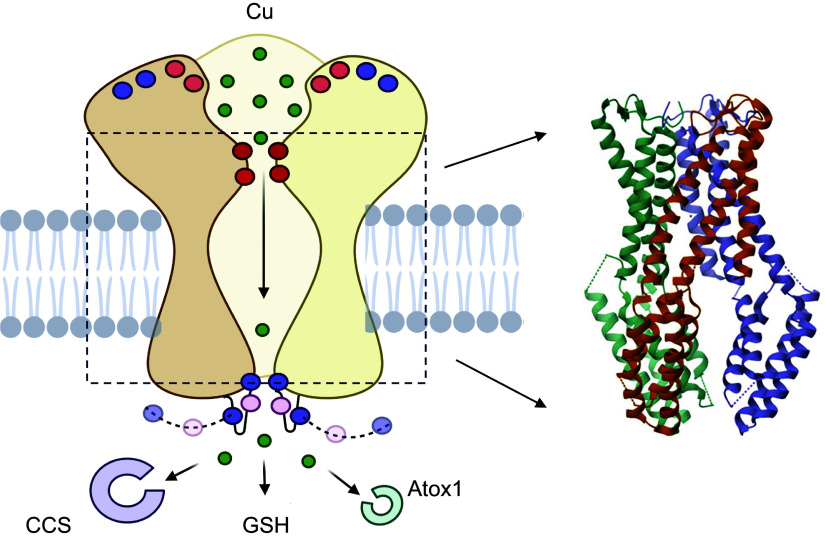
Cartoon depicting the cross-sectional view of copper (Cu) transporter CTR1 (*left*) and the experimental structure of the NH_2_ terminally truncated CTR1 (PDB 6M98; *right*). Ctr1 is a homotrimer. The extracellular domain of CTR1 contains clusters of Met (orange) and His (purple) residues that concentrate Cu and guide it toward the selectivity filter. The translocation pathway for Cu (green circles) is formed by the transmembrane domain 2 (TM2) of each monomer. Each TM2 contains an invariant MxxM motif (red circles) that is essential for activity. Cu binds to the first triad of Met residues (red circles at *top*) and then is handed to the second triad (red circles at *bottom*) before entering the intramembrane cavity. The COOH-terminal His-Cys-His motif (purple and pink circles) may regulate Cu release, transfer Cu to Cu chaperones (CCS and Atox1), and interact with VEGFR2 (210). Image created with BioRender.com, with permission.

In vitro studies of CTR1-mediated Cu transport revealed that it has a *K*_m_ of ∼3 μM and transports 5–10 Cu ions per protein per second (209). Given the CTR1 channel-like structure (FIGURE 3), this rate seems rather slow, and single-molecule studies may be needed to determine whether at every moment all CTR1 molecules at the cell surface are fully active.

CTR1 is a homotrimeric membrane protein with a copper translocation pathway located in the center of the trimer (211, 212). Each monomer has an extracellular NH_2_-terminal domain, which contains 10 Met residues and 8 His residues, arranged in clusters. With a total of 57 Cu-coordinating Met and His residues per trimer, the domain likely acts as a “Cu sponge” retrieving Cu from the environment (NCC/SCC) and directing it to the Cu translocation pathway (213, 214). The trimeric organization facilitates the reduction of Cu^2+^ to Cu^+1^ and stabilizes the reduced metal (214). Met residues also encircle the entry into the translocation pathway and serve as a “selectivity filter” for Cu^+1^ (FIGURE 3, *left*). Cu^+2^ is not transported by CTR1, whereas Ag^+1^, whose size and electronic structure are similar to Cu^+1^, competes with Cu^+1^ and diminishes Cu uptake. It was proposed that the metalloreductases of the STEAP family reduce Cu^+2^ to Cu^+1^ to facilitate Cu uptake (215). However, the reported effects were small, and further evidence for CTR1/STEAP interactions (either structural or functional) has not so far emerged. Recent data suggest that the NH_2_-terminal domain of CTR1 can reduce Cu^+2^ to Cu^+1^ on its own (213, 214).

Either in the blood or intracellularly, Cu is bound to various molecules and no free Cu exists (216, 217). Under these circumstances, the uptake of Cu is likely to be driven by a local concentration gradient formed when Cu binds at the Met/His-rich extracellular domain of CTR1. Downstream of the selectivity filter, the Cu translocation pathway of CTR1 is relatively wide and has no Cu-coordinating residues (FIGURE 3). The abundant cytosolic tripeptide glutathione [Glu-Cys-Gly (GSH)], which can bind Cu with a relatively low affinity (218), probably enters this wide cavity and buffers Cu within CTR1, thus protecting Cu(I) from disproportionation and facilitating Cu release from the selectivity filter and subsequent transfer to the cytosol. Pharmacological depletion of cellular glutathione is associated with a marked (50%) decrease in the rate of Cu uptake by CTR1 in agreement with this model (219).

In the cytosol, Cu binds to specific protein carriers (Cu chaperones, see details below) and possibly to other molecules (220), which have a higher affinity for Cu than glutathione (217, 218, 221). These cytosolic proteins form a pool of exchangeable copper, which can be visualized following the reduction of Cu-coordinating thiols and capturing the released Cu with a fluorescent sensor (217).

#### 3.1.2. Regulation of CTR1.

**FIGURE 4.**
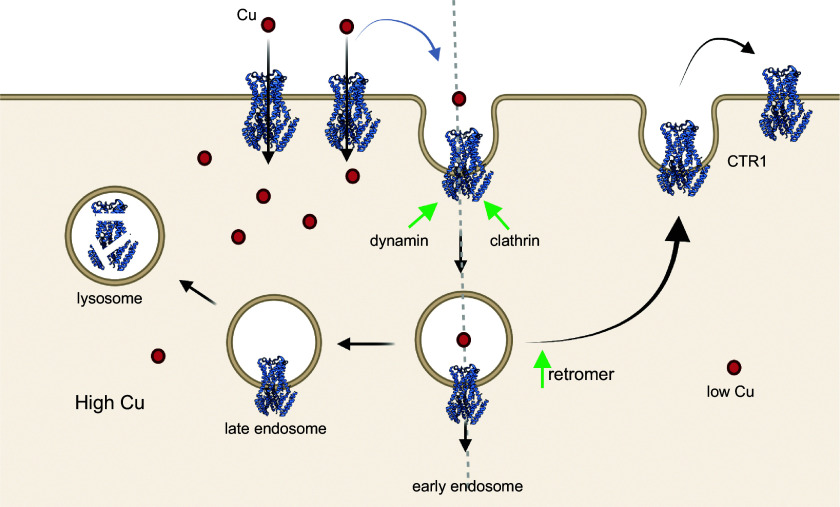
Copper (Cu) transporter CTR1 (SLC31A1) localization and traffic in a generic nonpolarized cell. In the vast majority of cells, CTR1 is located in the basolateral membrane and transfers Cu from Cu carriers in the blood into the cytosol. Intracellular Cu elevation triggers CTR1 endocytosis to early endosomes. Upon Cu depletion, CTR1 binds the retromer and returns to the plasma membrane. Upon prolonged Cu elevation, endocytosed CTR1 is degraded. Image created with BioRender.com, with permission.

In most cells, at steady state, CTR1 is located at the membrane facing blood (the basolateral membrane). Changes in Cu levels result in CTR1 cycling between this membrane and intracellular vesicles. Elevation of Cu causes CTR1 endocytosis from the plasma membrane, which prevents Cu overload. The invariant COOH-terminal His-Cys-His sequence of CTR1 plays an important role in this process (209). The internalization of CTR1 depends on clathrin and dynamin and progresses via the Rab5 and EEA1-containing vesicles (133). A decrease in Cu levels in the cytosol is associated with the trafficking of CTR1 from the vesicles back to the plasma membrane (FIGURE 4). This recycling process is facilitated by CTR1 interactions with the components of the retromer (222).

**FIGURE 5.**
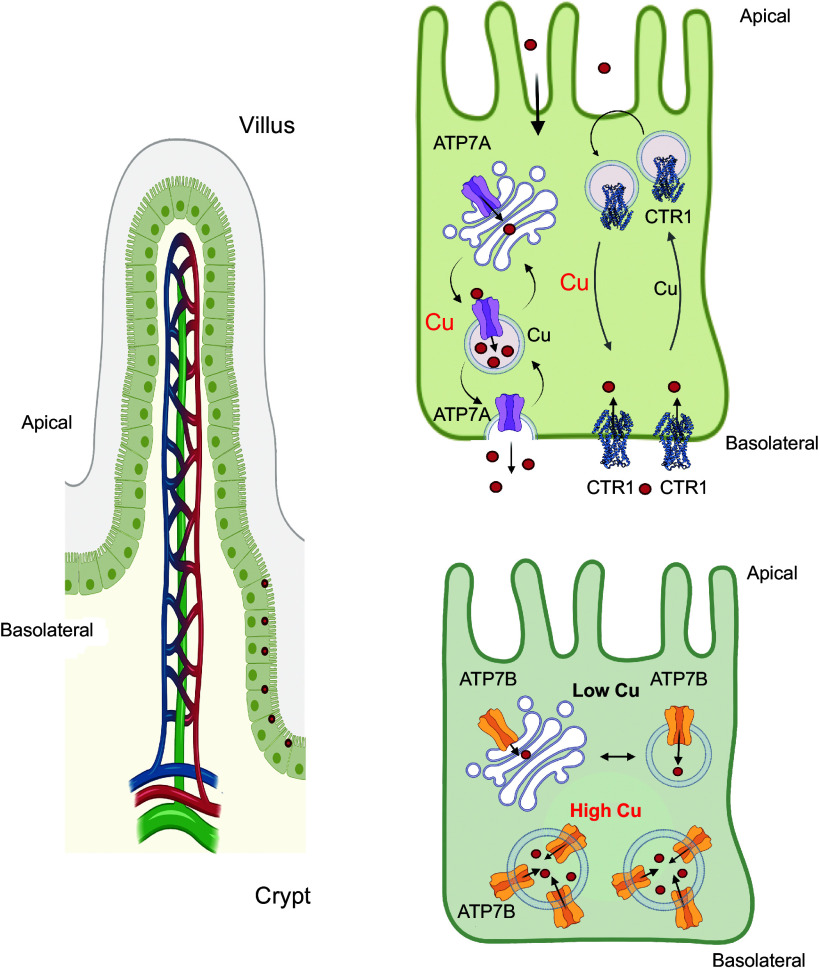
Copper (Cu)-dependent localization of Cu transporters in the small intestine. Cu (red circles) and Cu transporters (CTRs) are distributed unequally in the villi and the crypts. CTR1 and ATP7A, which are required for dietary Cu uptake, are enriched in the villi. Their expression in the crypt is low. ATP7B and Cu show an opposite pattern: highly enriched in the crypt and relatively low in the villus. The regulation of CTRs is also distinct. At steady state, the enteric CTR1 is targeted predominantly to the basolateral membrane. Cu depletion causes CTR1 trafficking from the plasma membrane to vesicles located in the vicinity of the apical membrane. ATP7A provides the major route for dietary Cu to enter the bloodstream from enterocytes. At steady state, ATP7A cycles between the *trans-*Golgi network (TGN) and endocytic vesicles, where it sequesters Cu for further export via vesical fusion. ATP7B is present predominantly in vesicles, although TGN localization is also observed. Upon Cu elevation, the abundance of ATP7B increases and the localization is uniformly vesicular. Image created with BioRender.com, with permission.

In the intestine (enterocytes), CTR1 localization is age dependent and more complex than in most other cells (FIGURE 5; Ref. 223). Convincing evidence exists for CTR1 basolateral localization when Cu is adequate and relocalization toward the apical membrane when Cu concentration is low (223–225). It was suggested that the apical trafficking reflects an additional role of CTR1 in the uptake of dietary Cu from the gut lumen (225). Genetic deletion of CTR1 in enterocytes causes systemic Cu deficiency, supporting the CTR1 role in dietary Cu acquisition (226). However, the CTR1-mediated Cu transport across the apical membrane and its contribution to the total Cu uptake from the gut lumen need to be directly measured, because the above-mentioned deletion of CTR1 does not prevent Cu entry into enterocytes (226).

In addition, the role of CTR1 in cell differentiation (227), the negative effects of CTR1 deletion on intestinal morphology (180), and the involvement of CTR1 during FGF, PDGF, EGF, and VEGF signaling (210, 227, 228) complicate interpretation of CTR1 deletion phenotype. Thus, although tremendous progress has been made, the function of CTR1 in the intestine and the role of CTR1 in growth factor signaling needs further study.

#### 3.1.3. The role of SLC11A2 (divalent metal transporter DMT1) and SLC7A5 in Cu transport.

The divalent metal transporter DMT1 (NRAM2 or SLC11A2) was suggested to play a role in Cu uptake from the gut lumen, under certain circumstances. The DMT1 preferred substrate is iron and, possibly, manganese, although distinct manganese transporters have recently been discovered (for review, see Ref. 229). The ability of DMT1 to transport Cu^2+^ was demonstrated in vitro (230), but the physiologic significance of DMT1-mediated Cu transport remains uncertain. Genetic inactivation of DMT1 in mice has no impact on the uptake of dietary Cu (231). Targeted inactivation of DMT1 in the intestinal epithelia led to a higher Cu uptake into the gut and iron deficit along with dysregulation of CP production, which is difficult to uncouple from iron misbalance (232). Thus, while DMT1 may be important “for optimal copper assimilation” (232), current evidence does not support the major role of DMT1 in the dietary Cu uptake into enterocytes. The existence of Cu transport mediated by anion exchange systems was suggested based on studies of polarized Caco-2 cells (233). The knockouts of candidate molecules would help to validate these suggestions.

Recently, it was reported that LAT1 (SLC7A5), a membrane transporter that supplies essential amino acids to the brain and other tissues, can also transport the CuHis2 complex (234). Although it is not yet clear how much this transporter contributes to overall Cu intake, the ability of LAT1 to transport CuHis2 has significant clinical implications (for example, as a delivery route for Cu-histidinate, a commonly used treatment for Menkes disease patients; Ref. 235). In another recent study, an entirely new mechanism of Cu entry was proposed: via Cu binding to hyaluronic acid and the intracellular entry of this complex via CD44 receptor-mediated endocytosis (164). While intriguing, this latter mechanism needs further examination because hyaluronic acid downregulates the cellular Cu-efflux system (164). The diminished Cu efflux may increase cellular Cu content without increasing Cu uptake (164).

### 3.2. Cu Transport from Intracellular Vesicles/Lysosomes

Within cells, Cu transport into the cytosol from endocytic vesicles requires CTR2 (SLC31A2). CTR2 appeared during evolution as a result of CTR gene duplication (236) and is structurally similar to CTR1. However, unlike CTR1, CTR2 lacks the NH_2_-terminal Met/His-rich domains and, on its own, does not have Cu transport activity. Inactivation of CTR2 in mice increases the cellular Cu content (237), and, in the brain, CTR2 deletion increases the abundance of Cu storage vesicles in the subventricular zone (238). These results support the role of mammalian CTR2 in Cu export from the intracellular compartments into the cytosol. How CTR2 works is unclear. Interestingly, CTR2 interacts with CTR1; this interaction is important for CTR2 stability (239). The interaction also results in a specific proteolytic cleavage within CTR1, which decreases the CTR1 transport activity (240). It is tempting to speculate that CTR2 interactions with CTR1 and/or other protein(s) are necessary to form a Cu transport-capable complex (perhaps similar to the CTR4/CTR5 heterotrimer in yeast; Ref. 241), but this possibility remains to be tested. Studies in *Schizosaccharomyces pombe* suggest that, in yeast, the metalloreductase Fre6 is required for Cu transport by CTR2 and that the expression of Ctr2 and Fre6 is coregulated by iron availability (242). In humans, CTR2 is abundantly expressed in mast cells and other cells with major secretory functions; CTR2 is especially high in serous glandular cells of the salivary gland. In these cells, the transcript levels of CTR2 greatly exceed those of CTR1, suggesting that CTR2 may have CTR1-independent partners and/or functions. The in vitro reconstitution of Cu transport activity by CTR2 may reveal the details of the CTR2 mechanism and allow comparison with the CTR1-mediated Cu transport. Such studies are ongoing (243).

Another intracellular transporter, SLC46A3, was recently proposed to import Cu from the cytosol to lysosomes, because SCL46A3 overexpression increased the lysosomal Cu content (244). A subsequent study, however, found that SLC46A3 was a proton-dependent transporter of steroid conjugates and bile acids (245). This finding suggests that the role of SLC46A3 in lysosomal Cu transport may be indirect and/or may be coupled to its function in cell lipid metabolism (244). Studies of another lysosomal lipid transporter associated with Niemann-Pick disease also found changes in tissue Cu levels/distribution in response to the transporter inactivation (246, 247). Given the involvement of lysosomes in Cu homeostasis (for review, see Ref. 248), the fact that bile acids can bind Cu (249, 250), and the fact that Cu misbalance dysregulates sterols biosynthesis (162), further studies of a relationship between lysosomal Cu and sterols may determine whether and how their transport is coregulated.

Cu transport into the mitochondria matrix is mediated by the Cu transporter SLC25A3 (251). SLC25A3 is located in the inner membrane of mitochondria and was originally identified as a phosphate transporter. Subsequent studies in yeast and mammalian cells provided convincing evidence that SLC25A3 also transports Cu^+1^ (60, 251). Defects in SLC25A3 expression reduce Cu levels in the mitochondria matrix and decrease the activity of cytochrome *c* oxidase (60). Characterization of cultured primary myoblasts during myogenesis demonstrated upregulation of SLC25A3 and the defects in cell proliferation and differentiation when SLC25A3 was deleted (252). These results link the increased Cu supply to mitochondria to higher energy demands during myocyte proliferation and functional maturation. Interestingly, Cu supplementation rescues the SLC25A3 knockouts, suggesting that additional Cu-transporting mechanisms may exist in mitochondria membranes (252). As described above, SCL25A3 is also involved in mitochondria flickering and regulation of fusion (173).

### 3.3. Intracellular Cu Shuttles Have a Growing List of Functions

**FIGURE 6.**
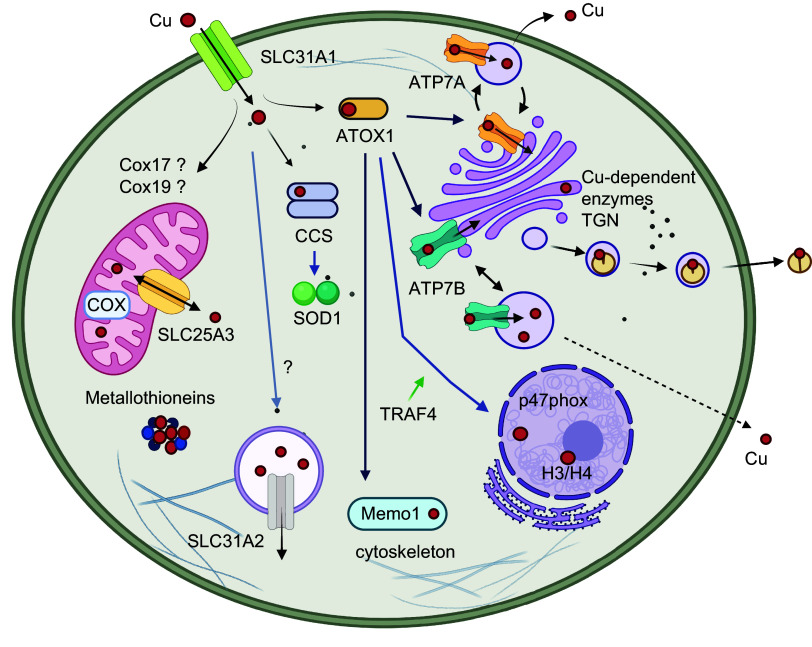
Copper (Cu)-distribution pathways within a generalized cell. Cu enters cells predominantly via the Cu transporter SLC31A1 (CTR1) and binds to cytosolic Cu chaperones (ATOX1, CCS, COX17, and COX19) and possibly other soluble proteins. CCS delivers Cu to cytosolic SOD1 via heterodimerization. ATOX1 exchanges Cu with the NH_2_-terminal domain of the Cu transporters ATP7A and ATP7B, which transfer Cu to Cu-dependent enzymes in the lumen of *trans*-Golgi network (TGN). When Cu is elevated, ATP7A and ATP7B move out of TGN to vesicles where they sequester excess Cu and eventually export it out of cells. ATOX1 also traffics to the nucleus in a TRAF4-facilitated fashion. ATOX1 can exchange Cu with mediator of cell motility 1 (Memo1), which binds to microtubules. Cox17 and Cox19 may be involved in Cu transfer to mitochondria but are not essential for this process. SLC25A3 is located in the inner membrane of mitochondria and maintains Cu content in the matrix. Mitochondria Cu chaperones are located in the inner membrane and facilitate Cu incorporation into cytochrome *c* oxidase (COX). Metallothioneins are induced in response to Cu elevation and bind Cu with high affinity to prevent toxicity (created with BioRender). Cu-accepting proteins in each of 3 intracellular Cu distribution pathways have specific structural features that facilitate Cu exchange with respective chaperones. Recent studies in yeast suggest that this classic model is somewhat simplistic and that a significant number of Cu-binding proteins exists in a cytosol forming an exchangeable “Cu pool” (220). Whether these proteins receive Cu from a chaperone, the Cu-glutathione complex, or other carriers is not yet clear.

Upon entering the cytosol, Cu binds to cytosolic proteins, which are collectively called Cu chaperones. Cu^+1^, which is imported into cells by CTR1, is highly reactive and, in the absence of ligands, can also disproportionate into Cu^+2^ and metallic Cu^0^. Binding to chaperones prevents these unwanted reactions and allows the delivery and transfer of Cu^+1^ to specific Cu-utilizing proteins throughout the cell (253). The classic model suggests three major destinations for Cu chaperones. One is the cytosol, which contains very abundant SOD1 as a primary Cu acceptor. Another is mitochondria with cytochrome *c* oxidase as a major Cu-requiring enzyme. The third is the secretory pathway, where most of the other Cu-dependent enzymes are produced. In this last route, Cu is not directly delivered by the chaperone to the enzymes. Instead, Cu is transferred to the intermediary, the ATP-driven Cu transporters ATP7A and ATP7B (see below), which then transport Cu to the lumen of the secretory pathway (FIGURE 6).

#### 3.3.1. Copper chaperone for superoxide dismutase 1 is a classic copper chaperone.

**FIGURE 7.**
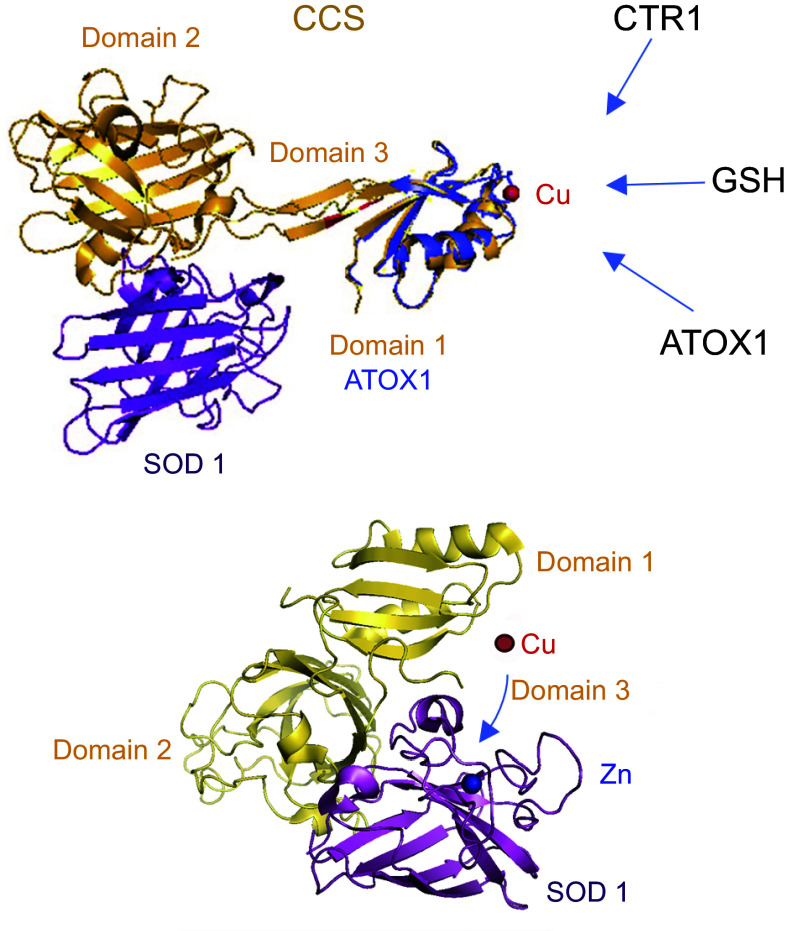
Human SOD1 in a complex with its copper (Cu) chaperone (CCS) in the elongated (*top*) and compact (*bottom*) conformations. CCS (yellow) has 3 domains. The similarity between the CCS domain 1 and ATOX1 (blue) is illustrated by the overlay of their structures. Domain 2 of CCS is structurally similar to SOD1 (purple), with which it heterodimerizes. Domain 3 carries cysteines that facilitate the formation of the disulfide bond in SOD1. In the elongated state (*top*; PDB 6FON), domain 1 of CCS can accept Cu from different sources (GSH, CTR1, and/or ATOX1). Following Cu binding to domain 1, CCS adopts compact information (*bottom*; PDB 6FP6). This brings domain 1 and domain 3 to the vicinity of the Cu entry site and leads to the insertion of Cu into SOD1, which has a zinc (Zn) atom already bound (256).

The copper chaperone for superoxide dismutase 1 (CCS) is a soluble cytosolic protein that interacts with the partially folded Zn-loaded SOD1 precursor (254). CCS then inserts, sequentially, Cu and the disulfide bond, both of which are essential for SOD1 stability and function (255). CCS binds Cu at the dedicated Cu-binding domain (domain 1) via characteristic Cys-xx-Cys motif and then heterodimerizes with SOD1 via domain 2, which is structurally similar to SOD1 (FIGURE 7). Cu is transferred to the catalytic site of SOD1 through cooperation between domain 1 and domain 3; this process is accompanied by the formation of an intramolecular disulfide bond in SOD1 (256). Cu deficiency upregulates CCS, apparently to maintain a Cu supply to SOD1. Curiously, CCS can also form a stable complex with CTR1, which can be displaced by apo SOD1 (257). Whether this interaction has consequences in vivo is unclear. Genetic deletion of CCS does not affect the rate of Cu uptake by CTR1 (219), although other, regulatory, roles for these interactions may exist.

In human cells, CCS is required for functional maturation of SOD1, whereas in other organisms, such as yeast, CCS can be substituted by other, currently uncharacterized molecules (258). Whether CCS is required for SOD1 maturation correlates with the oxygen dependence of disulfide-bond formation in SOD1, which differs in different organisms (259). Recent studies point to additional roles for CCS, including stabilization of protein conformation for the insertion of Zn cofactor (260) and Cu delivery to MEK1/2 kinase (128). Given the absence of structural similarity between SOD1 and MEK1/2, it would be interesting to determine how CCS accommodates and prioritizes these target proteins.

#### 3.3.2. Atox1 is a redox-sensitive Cu chaperone with multiple targets.

Atox1 is another extensively studied Cu chaperone with an increasing repertoire of functions. Mammalian Atox1 is a 7.5-kDa (68 amino acids) soluble protein, which has a ferredoxin fold and is structurally similar to the domain 1 of CCS (FIGURE 7). Atox1 has one Cu-binding site, formed by the Cys-xx-Cys sequence motif (261). Similarly to the CCS domain 1, the Atox1 Cu-binding site is located in the exposed flexible loop allowing two Cys residues within the Cys-xx-Cys motif to bind Cu and then exchange it with other proteins (120, 262, 263). The best-characterized function of Atox1 is to regulate Cu occupancy of the metal-binding domains (MBDs) in the regulatory domain of the Cu-transporters ATP7A and ATP7B (see Ref. 264 and below). The MBDs of ATP7A and ATP7B are structurally similar to Atox1. However, unlike the CCS-SOD1 pair, the Atox1 interactions with MBDs are transient and take place predominantly via Cu-binding sites during metal exchange (264). In vitro, domain 1 of CCS can substitute for Atox1 in Cu transfer reactions; however, in vivo, CCS does not substitute for Atox1, as evident from functional deficits of *Atox1*-/- mice (265, 266).

While the primary function of CCS is to insert Cu cofactor into the target protein SOD1, Atox1 not only transfers Cu to its targets but can also remove Cu from their binding sites. This property makes Atox1 an important regulator of protein Cu occupancy. Apo-Atox1 can retrieve Cu from the COOH terminus and the intracellular loop of CTR1, at least in vitro (263, 267). This activity may be responsible for the “reset” of the endocytosed CTR1 and its return to the plasma membrane when intracellular Cu concentration decreases (FIGURE 4). Similarly, Atox1-mediated Cu transfer to and from the regulatory domains of the Cu transporters ATP7A and ATP7B regulates their activity and possibly trafficking between the *trans*-Golgi-network (TGN) to endocytic vesicle (see FIGURE 6 and below). Recent studies found that Atox1 also can bind Zn forming dimer (268); the physiologic significance of Zn binding remains to be explored.

Atox1 is a redox-sensitive molecule, and its ability to bind Cu depends on the cytosolic ratio of reduced and oxidized glutathione (269). Neuronal cells use this property to increase Cu distribution toward the secretory pathway during cell differentiation (106, 270). A growing number of studies have implicated Atox1 in the regulation of DNA transcription, cell proliferation, and cell migration (271–273). Trafficking of Atox1 to the nucleus in response to various signals can be facilitated by other proteins, such as TRAF (FIGURE 6), and the enrichment of Atox1 at the cells’ leading edge was demonstrated (274–276). In addition to delivering Cu to ATP7A and ATP7B, Atox1 was shown to transfer Cu to Memo1 and potentially other proteins (120, 262, 263).

How ATOX1 responds to various signals at the molecular level and coordinates multiple activities is still unknown. Forced expression of Atox1 in the nuclei yielded a large number of affected transcripts (277). ATOX1 does not interact directly with DNA in vitro (275). However, it affects specific promoters, such as SOD3, in cell lysates (278), suggesting the involvement of other proteins in ATOX1-dependent regulation of transcription. Attempts to identify interacting partners using yeast complementation assay (279), immunoprecipitation (280), and APEX2 proximity labeling (281) produced a large collection of proteins with numerous nonoverlapping functions, making it hard to devise a cohesive mechanism of ATOX1 activity. Given these data, one wonders whether ATOX1 acts as a Cu chaperone in its original definition or, instead, serves as a cytosolic Cu-carrier that delivers Cu to many protein targets, with the exception of SOD1 and mitochondria. The upregulation of ATOX1 in several cancers (282) and its involvement in inflammatory vascularization (271) make ATOX1 a potential therapeutic target (for a recent review, see Ref. 282) and necessitate further studies to better understand ATOX1 functions and mechanism.

#### 3.3.3. Cu chaperones for mitochondria.

**FIGURE 8.**
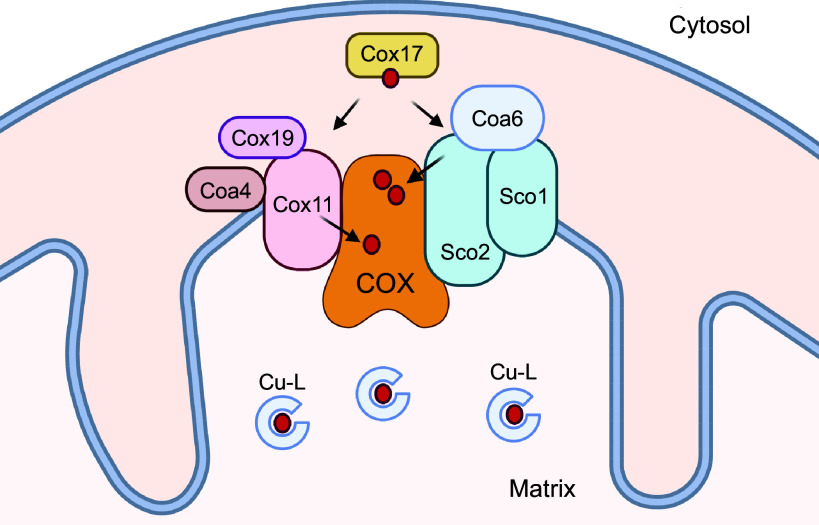
Schematic of chaperone-mediated Cu (red circles) delivery to cytochrome *c* oxidase (COX). Double red circles in COX are a CuA site and a single circle is a CuB site. Cu-L, low-molecular-weight Cu ligand in the mitochondria matrix. Image created with BioRender.com, with permission.

Although mitochondria are one of the most, if not the most, important destinations for intracellular Cu, which molecule(s) shuttle Cu from CTR1 to the mitochondria intermembrane space is still not understood. Within the mitochondria, several soluble and membrane-bound proteins work together in a series of redox-coupled reactions to bind and facilitate Cu delivery to cytochrome *c* oxidase Cu-binding sites (FIGURE 8; Ref. 59).

The soluble protein COX17 transfers Cu to the membrane-bound COX11, located in the inner membrane. COX17 together with COX19 and COA4 then facilitates the insertion of Cu into the CuB site of cytochrome *c* oxidase. COX17 also works with SCO1, SCO2, and COA6 to deliver copper to the CuA site of cytochrome *c* oxidase (59). Yeast and plants Cox11 have additional antioxidant activity that is independent of Cu chaperone function (283). Whether the mammalian COX11 has antioxidant activity is not known. Recently, *COX11* was identified as a disease-related gene. Mutations in COX11, similar to mutations in the mitochondria Cu chaperones SCO1 and SCO2, are associated with an infantile-onset mitochondrial encephalopathy (284–286) demonstrating the importance of COX11 for mitochondria function.

### 3.4. Cu Transport out of Cells by ATP7A and ATP7B

Cu entry into cells via CTR1 is an energy-independent process, whereas Cu export from cells requires ATP. Two structurally similar ATP-hydrolyzing and Cu^+1^-selective transporters, ATP7A and ATP7B, maintain Cu levels in the cytosol and facilitate Cu export (FIGURE 6). ATP7A is a primary Cu-efflux system in most tissues, except the liver, and it is essential for normal Cu homeostasis. In the intestine, ATP7A mediates dietary Cu uptake by exporting Cu into the bloodstream (FIGURE 5). ATP7A is also critically involved in the transfer of Cu across the brain barriers into the brain (see more below). Inactivation of ATP7A causes systemic Cu deficiency and the fatal neurodegenerative disorder Menkes disease (see sect. 3.6.1). Closely related ATP7B is a major Cu efflux system in the liver, where it facilitates the removal of excess Cu from hepatocytes into the bile. ATP7A is expressed in the embryonic liver but is absent in hepatocytes of the adult liver (223). In some polarized cells, such as epithelial cells of the choroid plexus (see below) and lactating mammary gland, ATP7B works together with ATP7A to export Cu across different membranes (287–289). In other tissues/cell types, such as enterocytes and noradrenergic neurons, ATP7A mediates Cu efflux, whereas ATP7B is targeted to endocytic vesicles and has Cu buffering and Cu storing functions; for a detailed review, see Ref. 81. Genetic inactivation of ATP7B causes Cu accumulation in the liver and other tissues and the potentially fatal metabolic disorder Wilson’s disease (see sect. 3.6.2.).

**FIGURE 9.**
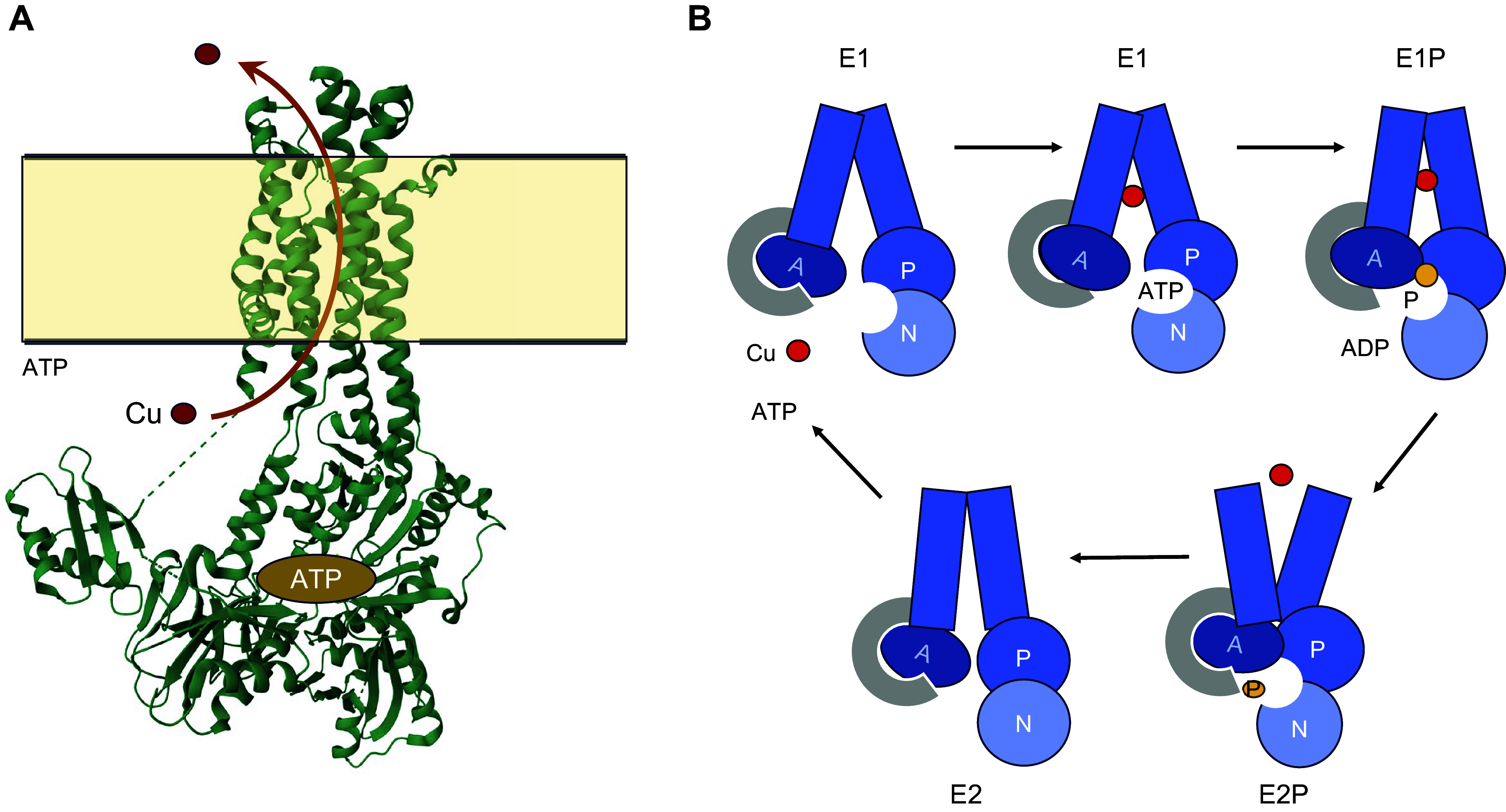
Current view of the structure and mechanism of Cu^+1^-transporting P-type ATPases. *A*: structure of a truncated ATP7B (PDB accession 7SI3; Ref. 294); for other conformers and the detailed discussion of structural changes during the Cu-transport cycle, see Ref. 293. *B*: cartoon illustrates the main steps of ATP-driven Cu transport: binding of ATP and Cu stabilizes the E1 conformation of the transporter allowing the ATP hydrolysis and a transient phosphorylation (P) of the invariant Asp residue within the P domain. N, N domain. Hydrolysis of this energy-rich acyl-phosphate bond is facilitated by the A domain and triggers the conformational transition resulting in the release of Cu at the opposite site of the membrane. The transporter then returns to the state that can bind Cu and ATP from the cytosol.

The function and mechanism of ATP7A and ATP7B have been extensively reviewed (81, 290–292); for the up-to-date summary of their structure-based mechanism, see Ref. 293. Briefly, these transporters belong to the P_1B_ family of metal-transporting ATPases that couple the energy of ATP-hydrolysis with Cu^+1^ transfer across the membranes. The ATP-dependent Cu transport relies on precisely coordinated interactions between the cytosolic domains (A, P, and N domain that are involved in ATP-binding and hydrolysis) and the transmembrane domain in which 8 transmembrane segments form the Cu translocation pathway (FIGURE 9).

The process begins with the binding of Cu^+1^ within the transmembrane part of the transporter along with the binding of ATP within the nucleotide-binding domain. The binding of both ligands facilitates the transfer of ATP g-phosphate to the invariant Asp residue in the cytosolic P domain. The acyl-phosphate bond is transient; the hydrolysis of this bond by water releases energy necessary for protein conformational transitions, which in turn allows Cu^+1^ to be transferred from the transmembrane site into the lumen of the secretory pathway for utilization by Cu-dependent enzymes (FIGURE 9).

Additional details on the structural organization of Cu-transporting ATPases and the proposed Cu transport mechanism can be found in Refs. 293–297. Although many aspects of ATP-dependent Cu transport are now better defined, many questions remain. How exactly is Cu transferred from the cytosol to the Cu translocation pathway (and the role of Atox1 in this process) remains a matter of debate. Despite significant advances in structural studies and the availability of AlphaFold, the full-length structure of either ATP7A or ATP7B is not yet available. A better understanding of the relationship between ATP7A and ATP7B is also needed, as many cells express both of these transporters at different ratios.

Unlike many other efflux transporters, neither ATP7A nor ATP7B work at the plasma membrane. Instead, their primary localization is intracellular, mostly within the distinct subcompartments of the TGN (298), Depending on a cell type, the organism’s developmental stage and the cell metabolic status of ATP7A and/or ATP7B can also be found in endocytic vesicles and/or in specialized compartments, such as melanosomes, phagosome, and autophagosomes (299–301). In the intracellular compartments, ATP7A and ATP7B facilitate the maturation of Cu-dependent enzymes (TABLE 1) by transferring Cu from the cytosol into the compartment lumen (FIGURE 6). In most cells under steady-state conditions, ATP7A is targeted to the TGN, where it activates DBH, AOC3, SOD3, LOX, LOX-like proteins, and possibly other Cu-dependent enzymes (53, 116, 302, 303). Tyrosinase receives its Cu from ATP7A in the melanosome (299), whereas neuronal peptidyl-a-monooxygenase recieves it in synaptic vesicles (304). ATP7B transfers Cu to CP in the TGN of hepatocytes (305). It was thought that Cu transfer from ATP7A or ATP7B to their respective protein targets did not require additional chaperones. While this could be true in some cases, a recent study revealed that ATP7A-mediated activation of LOX requires fibulin 4, which facilitates Cu-dependent formation of LTQ cofactor (112). In endocytic vesicles, ATP7A and ATP7B concentrate Cu either for further export or for storage. Here, the rate of Cu transport is accelerated by the higher acidity of the vesicle lumen, which is favorable for Cu release from the transporter.

Cellular Cu levels determine whether, at any given moment, ATP7A and ATP7B are involved in Cu transfer to Cu-dependent enzymes or in Cu buffering/export. Under basal Cu conditions, ATP7A and ATP7B are located in the distinct subcompartments of the TGN (298). In some cell types, predominantly vesicular localization was reported for ATP7B (180). Intracellular Cu elevation is thought to increase the Atox1-mediated delivery of Cu to ATP7A and ATP7B, increase Cu occupancy of their regulatory NH_2_-terminal domains, and facilitate ATP7A and ATP7B exit from the TGN (131). The distribution of Cu between ATP7A and ATP7B is probably determined by their relative abundance in respective cells, since their metal-binding domains (MBDs) in vitro behave similarly toward Atox1 (306, 307). Studies of ATP7B trafficking demonstrated that Cu-induced conformational changes within the regulatory domain allow for kinase-mediated phosphorylation of the transporter. Phosphorylation disrupts the inhibitory interdomain interactions, resulting in an increased Cu transport activity (131) as well as trafficking from the TGN. In addition to elevated Cu, trafficking of ATP7A and ATP7B can be triggered by hormonal signaling or cell differentiation signals (107, 158, 308).

Although ATP7A and ATP7B both traffic in response to Cu elevation, their final destinations differ. For example, in rat hippocampal neurons, ATP7B traffics toward the dendrites, whereas ATP7A moves toward the axons (309). In polarized epithelial cells, such as enterocytes, ATP7A and Cu-containing vesicles fuse with the basolateral membrane, whereas in hepatocytes ATP7B moves through the series of endocytic compartments to the apical membrane to export Cu (298). In the choroid plexus, the membrane polarity is inverse and ATP7A traffics to the apical membrane and ATP7B to the basolateral membrane (289). A recent study offers an excellent comparative analysis of similarities and differences in the mechanisms of ATP7A and ATP7B trafficking (298). Targeting of ATP7A to the basolateral plasma membrane is facilitated by the PLEKHA5, PLEKHA6, and PLEKHA7 proteins (310), whereas COMMD1 plays an important role in guiding ATP7B toward the apical membrane and recycling (311, 312). Deletion of COMMD1 impairs not only the trafficking of ATP7B but also the LDL receptor, whereas the trafficking of ATP7A is not affected (313, 314). Various other regulators of ATP7A/7B trafficking have been characterized (for detailed reviews, see Refs. 81, 292). How Cu(I) is buffered within the vesicles and in which form Cu is released into the extracellular milieu after vesicle fusion with the plasma membrane is unknown.

In addition to regulating Cu balance, trafficking of Cu transporters appears to have a signaling role by directly impacting the abundance and internalization of several receptors. VEGF is essential for angiogenesis and also plays an important role in wound healing, hematopoiesis, bone formation, and tumor biology. Recent studies uncovered a new functional relationship between VEGF and Cu transporters ATP7A and CTR1. ATP7A was shown to be essential for neovascularization, likely through its role in the activation of lysyl oxidase (315). In addition, the loss of ATP7A function in endothelial cells results in VEGFR2 degradation and inhibition of the VEGF-induced microvessels sprouting (315). CTR1 also enhances VEGF signaling (210). CTR1 appears to directly interact and coendocytose with VEGFR2 in response to VEGF; this process involves oxidation of cysteine residue within the CTR1 COOH terminus (210). This regulation is independent of CTR1 Cu transport function but critically dependent on the presence of COOH-terminal cysteine (210).

## 4. Cu DELIVERY AND UTILIZATION IN THE BRAIN

### 4.1. Distribution of Cu in Brain Regions

The Cu content of the brain (9% of the total body content) is among the highest in tissues after the liver and kidneys (316). In the brain, cells use Cu for such generic processes as energy production, iron metabolism (via ceruloplasmin and hephaestin, enriched in the basal ganglia), reactive oxygen species (ROS) scavenging, and angiogenesis (by SOD1 and SOD3). Cu is also required for more specialized functions: synthesis of neurotransmitters (by DBH and PAM), neuronal cell connectivity and adhesion, myelin production by oligodendrocytes, modulation of synaptic transmission, maturation of neuronal stem cells (317–321), and regulation of long-term potentiation (322, 323). The signaling role for Cu was demonstrated in the case of the NMDA receptor, the activation of which was shown to cause a release of Cu from the primary hippocampal neurons followed by inhibition of extrasynaptic GABA receptors (139). The list of neurologic and psychiatric conditions in which Cu misbalance has been observed is growing rapidly (29, 324–326) making a better understanding of brain Cu physiology increasingly important. Many disease-related findings are still phenomenological, but available mechanistic data highlight the critical involvement of Cu handling proteins in the pathogenesis and/or manifestations of several neurodegenerative diseases (29, 206, 327). For example, in the entorhinal cortex of schizophrenia patients, decreased levels of CTR1 were shown to correlate with other Cu-dependent events, such as expression of myelin basic protein (MBP) (328).

In some cases, a significant difference exists between the in vitro and in vivo findings with regard to Cu involvement in disease pathogenesis. Prion protein (PrPc), best known for the role of its mutant variant in fatal transmissible spongiform encephalopathies, is highly expressed in the brain. The nonpathogenic PrPc was implicated in numerous physiological processes, including regulation of Schwann cells-mediated myelination (329) and neurite outgrowth (for review, see Ref. 330). PrPc binds Cu^+2^ at the cellular surface; this promotes PrPc condensation and also protects cells against Cu toxicity (331). However, the in vitro experiments testing the effects of Cu on PrPc often used nonphysiologically high metal concentrations, and more recent studies in mice found only a minor role for Cu in prion disease pathogenesis (332). At the same time, mutation of Cu-binding residues in PrPc or Cu chelation diminishes the PrPc ability to regulate NMDA-mediated currents in hippocampal neurons lending support to a physiologically relevant PrPc-Cu relationship. Further studies of Cu homeostasis in the isogenic *Prnp^ZH3/ZH3^* mouse strain (333) may shed new light on the Cu-dependent functions of PrPc.

X-ray fluorescence microscopy (XRFM) and atomic absorption spectroscopy were used to examine the distribution of Cu throughout the brain (334–336). The dentate gyrus, the molecular layer of the hippocampus, the olfactory bulb, the locus coeruleus, and the subventricular zone in ventricles have higher Cu content than other brain regions (337, 338). Cu concentration is highest in the subventricular zone, in cells positive for glial fibrillary acidic protein (335). These cells contain intracellular structures called copper storage vesicles (CSVs) where Cu is highly concentrated (335). The abundance of CSVs and their Cu content increases with age (238, 335, 339, 340) and also following the deletion of CTR2 (SLC31A2) (240). Cu in CSVs decreases when metallothioneins are genetically ablated (339), suggesting that metallothioneins are responsible for Cu sequestration. It was suggested that the CSV-enriched cells represent Cu reservoirs for neuronal stem cells located nearby (339). As we discuss below, the amount of Cu entering an adult brain is low, and the availability of Cu reserves is essential for tissue repairs in case of injury. Experiments with intracerebroventricular infusion of Cu chelators demonstrated that Cu limitation impacted the number of newborn neuroblasts in the olfactory bulb and the expressions of mRNAs for proteins that regulate adult neurogenesis, including, Shh, Dlx2, and Slit1 (321). How brain parenchyma and/or stem cells communicate their Cu status and their Cu needs to the CSV-containing cells is a fascinating question for future studies.

In addition to the nonuniform distribution of Cu throughout the brain, morphologically complex cells, such as neurons, have an uneven distribution of their intracellular Cu. In neurons, Cu is more concentrated in cell bodies, dendrites, and synapses and is less abundant in axons (341). In the spine of synaptic buttons, Cu is concentrated in the vicinity of actin (167, 169). Identifying the molecular basis of nonuniform Cu distribution is likely to open a new chapter in understanding of regulatory functions of Cu in cells.

### 4.2. Cu Entry into the Brain Is Age Dependent

Adequate Cu supply is essential for postnatal brain maturation. Many developmental processes take place during this time: among them are the establishment of cell connectivity, myelination, increased energy expenditure, and catecholamine production. These processes, directly or indirectly, depend on Cu availability and are negatively affected by Cu deficit (342–344). Menkes disease patients, who lack the functional Cu transporter ATP7A and have impaired Cu delivery to the brain, show hypomyelination, abnormal vasculature, catecholamine misbalance, defects in axonal arborization and synaptogenesis, neuronal degeneration, and other pathologies (see Refs. 92, 345, 346 and below). These patients suffer from marked developmental delays and die in early childhood (347, 348). Similarly, the mottled-brindled mice, an animal model of Menkes disease, do not survive beyond postnatal *day 14*, unless rescued by Cu supplementation using Cu-elesclomol (ES) (349) or the adeno-associated virus (AAV)-mediated *Atp7a* gene transfer together with Cu-histidine injection into the brain ventricles (350). Dietary Cu deficit during development has a particularly significant negative effect on the maturation of the dentate gyrus and hippocampus (351).

In agreement with the requirements for Cu during development, the rate of Cu influx into the brain is highest during the neonatal period; in rodents, this period is between *day 7* and *day 14* after birth (352). Studies using the glyoxalbis-*N*(4)-methyl-3-thiosemicarbazonato-Cu(II) complex (^64^Cu-GTSM) found comparable Cu uptake by all brain regions with only a 15–20% higher influx into the thalamus, midbrain, cerebellum, and pons (353). Therefore, an uneven Cu distribution in the adult brain at steady state is likely a reflection of region-specific differences in the abundance of Cu-containing enzymes and/or presence of Cu-rich compartments, such as mitochondria or CSVs. For example, locus coeruleus, one of the Cu-rich regions, has abundant expression of Cu-containing DBH (116, 117), whereas the CSV-containing subventricular area is rich in metallothioneins. Cu uptake by specific brain regions can be increased by aggressively proliferating tumors, such as glioblastoma multiforme (354).

After the neonatal period in humans and animals, the rate of Cu entry into the brain decreases dramatically (176, 352). PET/CT studies of healthy human subjects found that within 1.5–6 hours of intravenous injection of Cu^67^, the adult brain absorbs only 0.2–0.3% of injected Cu, whereas other extrahepatic tissues absorb 45–50% of the dose during the same time (176). Despite the low rate of Cu uptake, with age Cu accumulates in the brain, suggesting that the export of Cu from the brain is negligible (340). ICP-MS analysis of brain regions of C57BL/6 mice between *day 81* and *day 560* after birth identified striatum and cerebellum as brain regions with the largest Cu increase during this period, 115% and 63% increase, respectively, whereas Cu content of the cortex and hippocampus did not change during the same period (355). Further studies, using mice heterozygous for *Ctr1*, demonstrated that Ctr1 contributes to the region-specific age-dependent Cu elevation but is not solely responsible for it (355).

### 4.3. Neurons and Glia Have Distinct Cu Homeostasis

**FIGURE 10.**
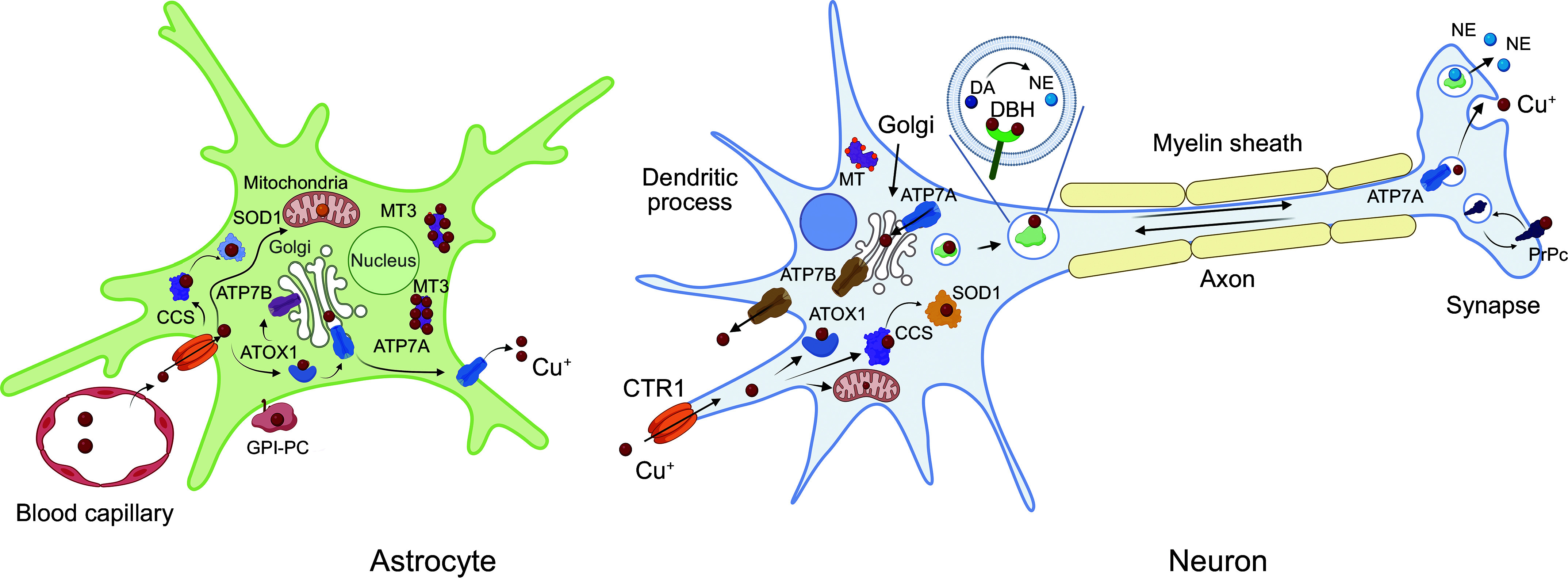
Copper (Cu)-handling machinery of neurons and astrocytes. Astrocytes play an important role in brain Cu homeostasis, as they are highly effective in storing and releasing Cu. Cu enters the brain through blood capillary of the blood-brain barrier and is taken up by astrocytes via the Cu transporter CTR1. Metallothionein MT3 and GSH are abundant in astrocytes and facilitate Cu buffering and storage. ATP7B may be involved in Cu transfer to the GPI-anchored ceruloplasmin, which is relatively abundant in astrocytes (8). ATP7A exports Cu, which is taken up by neighboring neurons via CTR1. Different neurons express ATP7A and ATP7B at different ratios (317). ATP7A and ATP7B are involved in the biosynthesis of Cu-dependent enzymes, removal of excess Cu, and sequestering Cu in vesicles for Cu storage or signaling. Cu-dependent enzymes, like dopamine-β-hydroxylase (DBH), are synthesized in specific types of neurons, where they produce neuromodulators that are packaged in secretory granules and then released in response to signaling events (CCS, Cu chaperone for SOD1; DA, dopamine; NE, norepinephrine). In response to changes in Cu levels, ATP7B traffics toward the dendrites (somatodendritic polarity). ATP7A is present in the cell body and traffics toward the axons in response to Cu elevation; ATP7A is also located at the synapse where it releases Cu in response to NMDA receptor signaling (139). Cu-binding protein PrPc (prion) is highly expressed at the synaptic cleft and is endocytosed in the presence of high Cu. Image created with BioRender.com, with permission.

Information about Cu physiology of neuronal and glial cells is very limited; the emerging data indicate a very significant difference in the Cu homeostatic mechanisms between neurons and astrocytes (356). Each of these cell types expresses key components of Cu homeostatic machinery, such as ATP7A, ATP7B, CTR1, and ATOX1 (Human Protein Atlas and Ref. 357), which respond to changes in Cu levels (FIGURE 10).

Cultured astrocytes treated with Cu show trafficking of ATP7A from the *trans*-Golgi-network (TGN) to the plasma membrane, in agreement with its role in the export of excess Cu (358). Astrocytes are the first parenchymal cells to receive copper that crosses the blood-brain barrier (BBB) (359). Early studies suggested that in addition to CTR1, astrocytes may have additional Cu uptake mechanisms, but their molecular identity remains unclear (360). Cu also regulates astrocyte metabolism. Cu treatment of cultured astrocytes increases glucose consumption and lactate release (361). This change in glycolytic flux is not due to mitochondrial impairment; it reflects the upregulation of transcription and/or translation of genes associated with glycolysis and glucose uptake (361). Although astrocytes can uptake and store larger amounts of copper than neurons, they are more sensitive to a copper-bound form of ionophore elesclomol (362) (see more on elesclomol below).

Oligodendrocytes are specialized glial cells that produce myelin sheaths. A single oligodendrocyte can myelinate ∼80 axons facilitating their conductance and providing metabolic support (363, 364). The requirement for Cu in maintaining myelin structure was reported as early as 1959 (365). For many years, Cu chelator cuprizone has been used to trigger reversible myelin loss. Whether the loss of myelin is caused by Cu depletion or toxicity of the Cu-cuprizone complex has been debated (366). To resolve this issue, recently, Cu measurements were carried out in various brain regions including corpus callosum, a major site of cuprizone-induced demyelination. These studies found a statistically significant decrease of Cu in a soluble fraction of tissue homogenates from the corpus callosum but not in other brain regions (320). The membrane-permeable Cu^II^(atsm) complex reversed both the Cu loss and demyelination, supporting the role of Cu in myelination (320). In further support, in humans and animals, copper deficiency is associated with characteristic symptoms of spinal cord demyelination (22, 148, 367, 368).

Both the direct effects of Cu on myelin (through the binding to and regulation of stability of myelin basic protein (MBP; Ref. 369) and the indirect effects of Cu chelation on cell lipid content have been reported (370). The dependence of iron metabolism on Cu availability led to the suggestion that Cu-dependent change in iron levels could affect the synthesis of myelin proteins and lipids (371). Cu released by astrocytes in response to signaling by neurotrophin receptor TrkB was suggested to cause oligodendrocyte loss, although the mechanism is still unclear (372). To add to the complexity, Cu-dependent myelination correlates with the expression of insulin-like growth factor in astrocytes adjacent to the site of myelin loss. Systematic studies of Cu metabolism in healthy oligodendrocytes during their functional maturation and establishment of cell-cell contacts may provide a clearer picture of how Cu is used by oligodendrocytes to perform their functions.

Information about spatiotemporal expression of Cu transporters in a brain is limited. Different expression of Cu transporters in various neuronal and glial cells is apparent from the single-cell sequencing studies (317) and may change during brain development. For example, in neonatal mice, Purkinje neurons of the cerebellum express Atp7b, whereas Bergman glia expresses Atp7a; subsequently, the expression of Atp7a in Purkinje neurons increases significantly (373, 374). In general, Atp7a expression coincides with neuronal maturation and increases during postnatal synaptogenesis. Distinct cellular localization and functions for ATP7A and ATP7B were reported in SHSY-5Y cells, a neuronal cell model (106, 116). In these cells, ATP7A is targeted to the TGN and is upregulated to accommodate increased expression of PAM and DBH during differentiation, whereas ATP7B buffers Cu in vesicles and is unchanged during differentiation (116). Downregulating ATP7A or chelating Cu prevents DBH secretion from SHSY-5Y cells, whereas inhibiting ATP7B stimulates DBH secretion (116).

### 4.4. The Blood-Brain Barrier and the Blood-Cerebrospinal Fluid Barrier Contribute to Brain Cu Uptake

**FIGURE 11.**
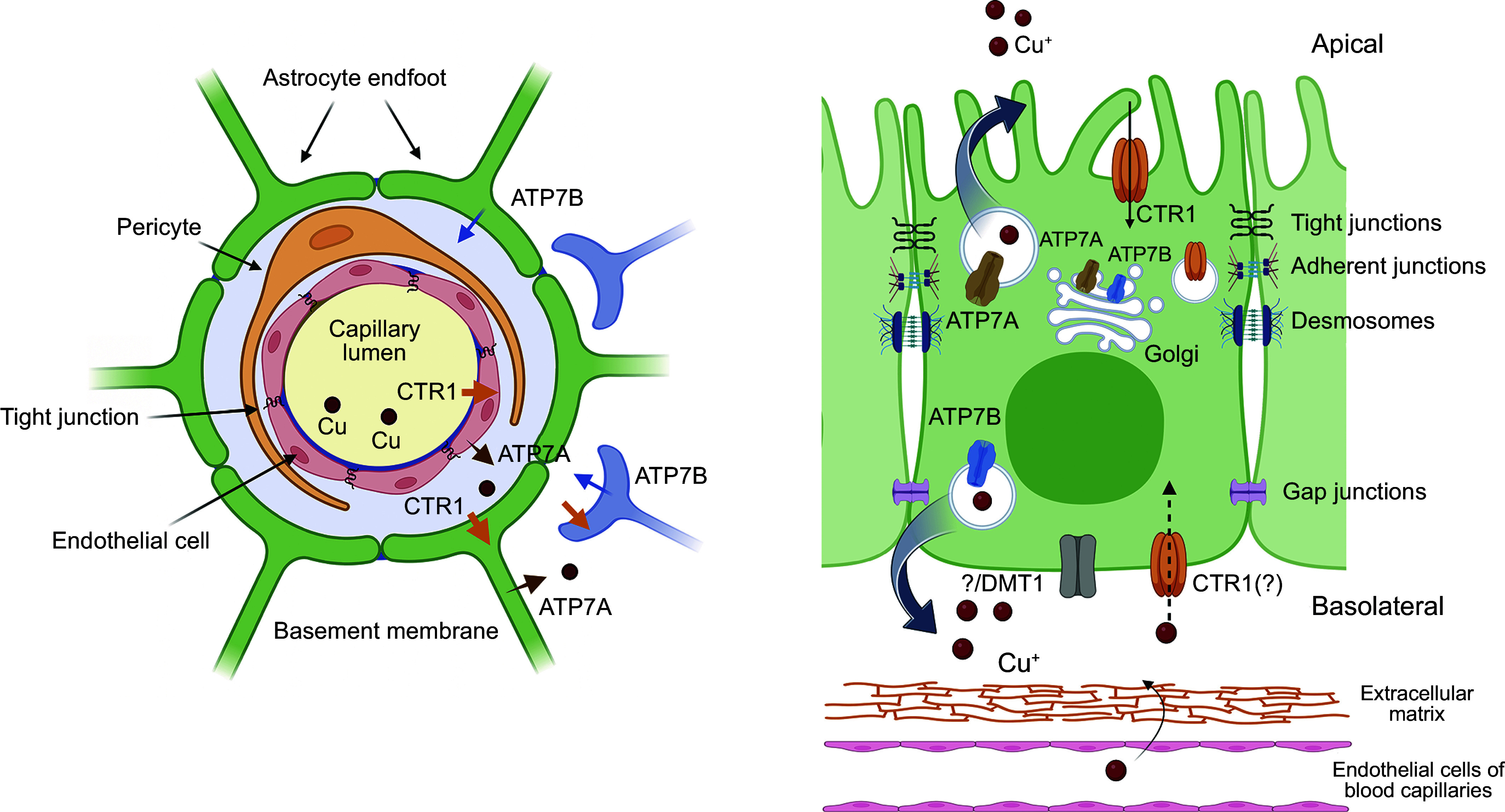
Copper (Cu) transporters in the brain barriers. *Left:* blood blood-brain barrier is composed of endothelial cells of the capillaries surrounded by astrocytic end feet, neurons, and pericytes. The endothelial cells express the Cu transporters CTR1, ATP7A, and ATP7B. The CTR1 retrieves Cu from the blood and ATP7A exports Cu toward brain parenchyma through the abluminal surface. Astrocytes import Cu through CTR1 and export Cu through ATP7A. *Right:* epithelial cells of choroid plexus (ChPl). The basolateral membrane of ChPl epithelia faces the blood capillaries, whereas the apical membrane is exposed to the cerebral spinal fluid. Both ATP7A and ATP7B are expressed in ChPl epithelia. Upon Cu elevation in ChPl, ATP7A traffics toward the apical surface allowing Cu entry into the cerebral spinal fluid/brain parenchyma, whereas ATP7B may export Cu through the basolateral membrane. CTR1 was detected at the apical surface, and its presence at the basolateral membrane needs to be examined further. Image created with BioRender.com, with permission.

Current data suggest that Cu enters the brain from the peripheral circulation through the blood-brain barrier (BBB) and the blood-cerebrospinal fluid (CSF) barrier (BCB). BBB comprises vascular endothelial cells, connected by tight junctions and surrounded by pericytes, the astrocytes end-feet, and neuronal cells (FIGURE 11, *left*). The BCB structure is simpler: the barrier consists of the cuboidal polarized epithelial cells connected by tight junctions and arranged around the blood capillaries (FIGURE 11, *right*). The apical side of the choroid plexus epithelium faces the cerebrospinal fluid (CSF), whereas the basal surface is oriented toward the endothelial cells of the vasculature. The relative contribution of BBB and BCB to Cu transfer to the brain and how this contribution changes during brain development are not fully understood. Perfusion of brain ventricles with ^64^Cu found that Cu uptake was highest by choroid plexus (ChPl), followed by blood capillaries, brain parenchyma, and CSF (375). It was proposed that Cu enters brain parenchyma through BBB and returns back to circulation via BCB (375). However, accumulating data suggest a more complex picture.

Two transporters, ATP7A and CTR1 (SLC31A1), are essential for delivering Cu to the brain (FIGURE 11). Patients with mutations in CTR1 show severe neurological dysfunction, cerebral atrophy, and ventriculomegaly, which reflect insufficient Cu supply, and they die in infancy (206). CTR1 is expressed in both brain barriers, and its expression increases in response to Cu deficiency (223, 376). Upregulation of CTR1 in vasculature supports its role in facilitating Cu transfer from the bloodstream to the cells of brain barriers: to correct Cu deficit in brain parenchyma. However, the epithelial cells of the choroid plexus (BCB) express Ctr1 (and Atp7a, see below) at significantly higher levels than the endothelial cells of BBB. In fact, CTR1 (SLC31A1) is among the most abundant transporters present in choroid plexus epithelia (377). In epithelial cells of the choroid plexus, CTR1 was found at the apical (CSF facing) membrane, in agreement with CTR1 removing Cu from the CSF (117, 223). This observation explains the high entry of Cu into the choroid plexus upon infusion of ventricles (see above) but raises questions about the mechanism of Cu import into the choroid plexus via the basolateral membrane. Furthermore, the diet-induced Cu deficiency increases Ctr1 abundance at the apical (CSF-facing aspect) of BCB (223). If CTR1 removes Cu from the brain, it would be counterproductive under conditions of Cu deficit.

**FIGURE 12.**
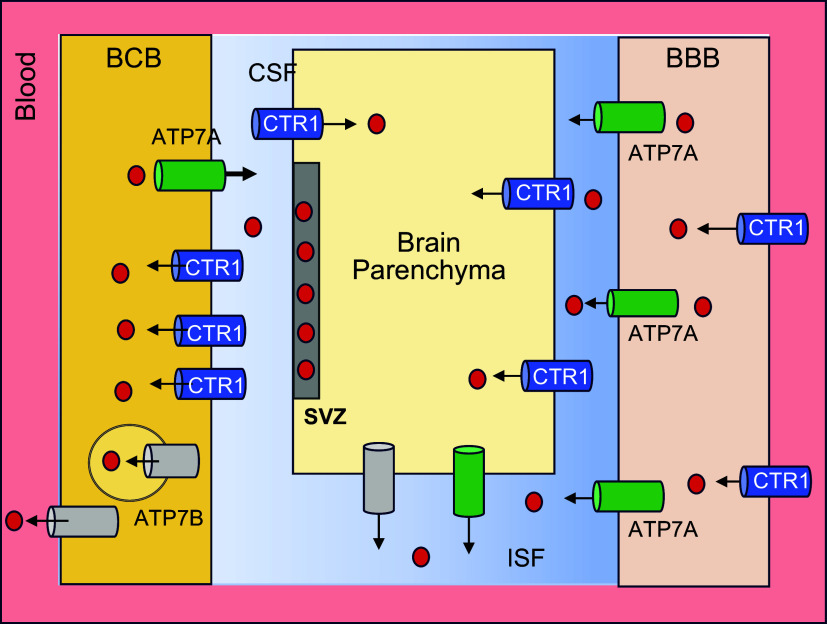
Potential roles of blood blood-brain barrier (BBB) and blood-cerebral spinal fluid (CSF) barrier in the entry of copper (Cu) into the brain. Cu (red circles) is taken from the blood predominantly by the Cu transporter CTR1 located in BBB (blue cylinder) and then exported out of BBB cells into the interstitial fluid (ISF) by ATP7A (green cylinder). Some Cu is taken by the parenchyma cells (via CTR1), while excess Cu enters the epithelium of the choroid plexus via CTR1 for storage. ATP7B (gray cylinder) may store Cu in vesicles and/or export Cu back into circulation via basolateral membrane. ATP7A transports to the CSF. Cu entering the brain parenchyma from CSF is stored in Cu storage vesicles in the subventricular zone (SVZ).

An alternative scenario is that BBB is responsible for Cu entry, whereas BCB controls the amount of Cu circulating in the interstitial fluid (FIGURE 12). High expression of CTR1 at BCB, which has approximately one-half of the BBB surface, ensures that all excessive Cu that may enter interstitial fluid via BBB is absorbed by the choroid plexus. In this scenario, the choroid plexus acts as a Cu-buffering tissue, which either stores excess Cu or exports Cu based on metabolic needs. When Cu is present in excess, it is removed by ATP7B. Under conditions of insufficient Cu supply to the brain by BBB, ATP7A in BCB utilizes the Cu pool stored in the BCB epithelial cells and exports Cu to CSF to increase Cu availability to brain parenchyma.

The following data support this model. ATP7A is expressed in both BBB and BCB (375, 378) and thus contributes to Cu transport through these barriers. The importance of ATP7A in Cu delivery is evident from the Menkes disease phenotype, where inactivation of ATP7A results in brain Cu deficit, neurodegeneration, and death in early childhood (375). Mottled brindled mice have a two-amino-acid deletion in the transmembrane segment 4 of Atp7a and recapitulate major aspects of human Menkes disease, including brain Cu deficiency (379). Characterization of these mice revealed a compensatory upregulation of ATP7A in the endothelial cells of microvessels and increased association of astrocytes within the blood-brain barrier, whereas ATP7A levels in choroid plexus and ependymal cells were not changed (380). Studies of another animal model of Menkes disease, macular mice, found Cu accumulation within the BBB components, i.e., astrocytes and endothelial cells in agreement with the important role of ATP7A-mediated efflux of Cu from the cells of BBB (381–383).

At the same time, several lines of evidence illustrate the role of ATP7A in transferring Cu to the brain via BCB (FIGURE 12). In the choroid plexus, Cu elevation causes trafficking of ATP7A to the apical membrane of epithelial cells, a process commonly associated with Cu export (289). The AAV-mediated delivery of Atp7a transcript to ChPl prevents mortality of *Atp7a*-/- mice, indicative of Cu transfer to the brain (350, 384). The disease manifestations in the Menkes disease patient with a somatic mosaicism for the inactivating ATP7A P1001L mutation further support the importance of ATP7A-mediated Cu export from ChPl. In this patient, cells of ectodermal origin (ChPl epithelia) had more frequent ATP7A mutation (and therefore more impact on Cu transport) than cells of mesodermal origin (endothelial cells of BBB; Ref. 385).

The role of Atp7b in Cu entry into the brain is not well defined. Increasing Cu in the CSF of adult mice triggers Atp7b trafficking toward the basolateral side of ChPl cells, suggesting Atp7b’s role in Cu export from the brain. Studies of postnatally developing *Atp7b*-/- mice found Cu entrapment in ChPl, downregulation of Ctr1 (in agreement with intracellular Cu elevation), and upregulation of Atp7a, possibly in response to transient Cu deficiency in brain parenchyma (117). Taken together, the available data point to ChPl epithelia serving as a rheostat that controls precisely the brain Cu content. In Wilson’s disease (WD) patients, cerebrospinal fluid (CSF) copper (Cu) content is elevated, reflecting Cu accumulation in the central nervous system (CNS). Studies have shown that with chelation therapy, CSF Cu levels gradually normalize over a period of approximately 2 years (386). The prolonged timeframe required for Cu normalization indicates that Cu transport and clearance from the CNS are tightly regulated processes. Understanding these mechanisms is crucial for refining therapeutic strategies that effectively lower Cu levels in the CNS and alleviate neurological symptoms in WD patients.

## 5. DISORDERS OF COPPER IMBALANCE

### 5.1. Pathophysiology of Menkes Disease and Emerging Treatments

Menkes disease and Wilson’s disease are the two best-characterized disorders of Cu homeostasis. Menkes disease is an X-chromosome-linked disorder, caused by missense mutations or chromosomal rearrangements in the *ATP7A* gene that lead to inactivation of the Cu transporter ATP7A. Loss of ATP7A function results in the entrapment of Cu in the intestine, impaired delivery of Cu to the brain, and a systemic Cu deficiency (387). Menkes disease is rare. Earlier estimates of the disease frequency found the incidence to be 1:254,000 births in European countries (388) and 1:354,507 in Japan (389) based on identified cases, although a higher frequency was reported for Australia (390). A more recent study using the Genome Aggregation Database (gnomAD) and frequency of pathogenic *ATP7A* alleles predicted a much higher occurrence of Menkes disease in the US population: 1 in 34,810 live male births (391). This result suggests that Menkes disease may be underdiagnosed, potentially due to miscarriages and early mortality (391). In addition to classic Menkes disease, where the function of ATP7A is entirely or almost entirely lost, less deleterious mutations in ATP7A are associated with milder phenotypes, such as occipital horn syndrome and distal motor neuron myelopathy (392).

In classic Menkes disease, symptoms appear within 6–8 weeks after birth. Patients suffer from poor temperature control, seizures, and neuronal degeneration and have low levels of ceruloplasmin, “kinky” hair, dilated and tortuous vasculature, and connective tissue abnormalities (393). They fail to thrive and typically die by the age of 3 (for review, see Ref. 394). Early Cu supplementation (within 2 months after birth) using injections with Cu-histidinate may significantly delay the development of symptoms. However, this treatment does not provide a cure, and the beneficial effects as well as toxicity vary (381, 395, 396). Menkes disease pathology illustrates the essential role of ATP7A in supplying Cu to tissues and the significance of Cu for brain development and function. The detailed analysis of disease pathogenesis has been difficult due to the early mortality of humans and experimental animals. Proteomes of cultured fibroblasts isolated from Menkes disease patients and their nonaffected family members were compared to identify proteins and pathways dysregulated in affected individuals (397). While these in vitro studies are useful, their relevance to the disease mechanism is somewhat tenuous, as the systemic effects of Atp7a inactivation markedly modify the consequences of Atp7a inactivation in specific tissues (398).

**FIGURE 13.**
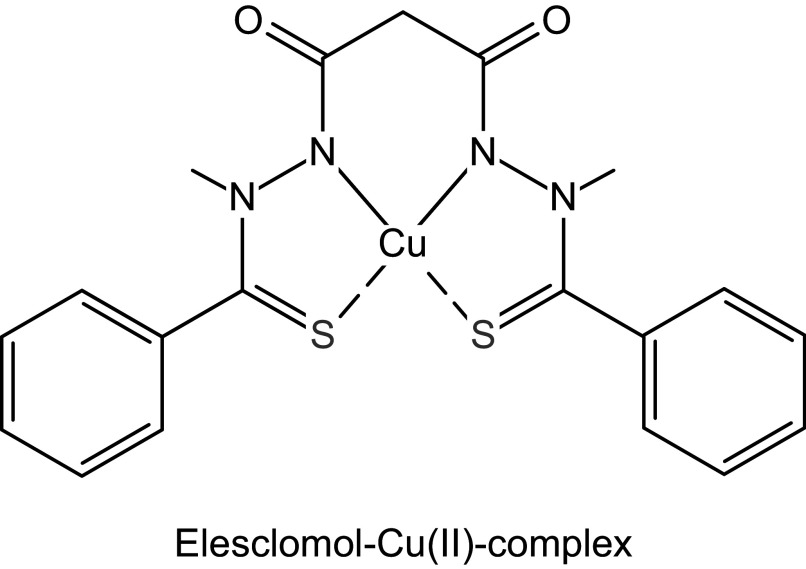
Structure of elesclomol in a complex with copper (Cu).

The most significant recent advance in mechanistic understanding and treatment of Menkes disease came with the discovery that a small molecule elesclomol (ES) in a complex with Cu (FIGURE 13), alleviates mortality and diminishes pathology in the mouse model of Menkes disease (349). Elesclomol (ES) is a molecule developed by Synta Therapeutics in collaboration with GlaxoSmithKline for the treatment of metastatic melanoma (for details, see below). Subsequent studies demonstrated that ES can restore mitochondria copper content and mitochondria respiratory function in the cellular and animal models of Cu deficiency (58). ES acts as a Cu ionophore: it delivers Cu to mitochondria (and to a lesser degree to a secretory pathway) independently of Cu transport machinery (399, 400). In addition to increasing Cu levels, treatment with ES-Cu increases cellular and mitochondrial Fe (400).

The markedly improved survival of the mouse model of Menkes disease treated with ES-Cu (349) demonstrated a major role of mitochondria in Menkes disease pathogenesis as well as a relatively higher importance of ATP7A activity in brain barriers compared to brain cells. The results explained a mild phenotype of mice with Atp7a deleted selectively in motor neurons compared to animals with Atp7a inactivated in whole body (398) and suggested potential clinical applications for the ES-Cu complex. Since elesclomol has an acceptable safety profile (401, 402), ES-Cu has recently been tried for the emergency treatment of Menkes disease patients in Europe. Based on the initial results, the European Medicines Agency designated ES-Cu as “an orphan medicinal product” for the treatment of Menkes disease. This development offers a glimpse of hope to Menkes patients and their families, although many challenges remain. Similarly to Cu-histidinate, ES-Cu does not cross brain barriers and has to be employed almost immediately after birth for its beneficial effects to be apparent (346, 349). In addition, Cu-delivering drugs can worsen kidney function, because in Menkes disease patients, kidneys, unlike most other tissues, accumulate Cu, which leads to toxicity. A recent finding that the endogenous metabolite a-lipoic acid can decrease Cu toxicity in cultured *Atp7a*-/- cells by improving cellular redox environment (137) raises a question whether a-lipoic acid can be used to improve kidney function in Menkes disease patients.

### 5.2. Wilson Disease

Wilson’s disease is an autosomal recessive disorder of copper overload, caused by mutations in the gene encoding the ATP-driven Cu-transporter ATP7B (115). The reported disease incidence varies significantly (from 1:5,000 to 1:100,000) depending on the world population and the method of analysis. The compound heterozygous nature of WD and the ability of different ATP7B protein variants to dimerize and modify properties compared to the respective homozygous mutants (403, 404) may explain, in part, the vast variety of disease manifestations and times of disease onsets. Other modifying factors include interdependence of Cu homeostasis and energy metabolism, natural genetic variants of such proteins as ApoE, and the tissue abundance of copper-chelating metallothioneins (405–408). Recent studies also uncovered an inhibitory effect of elevated Cu on cellular Se levels and selenoprotein production (137). This finding raises the question of whether the well-known ability of elevated Cu to cause oxidative stress can, at least in part, be ascribed to the diminished levels of selenoproteins, which are major contributors to cellular redox balance (409).

**FIGURE 14.**
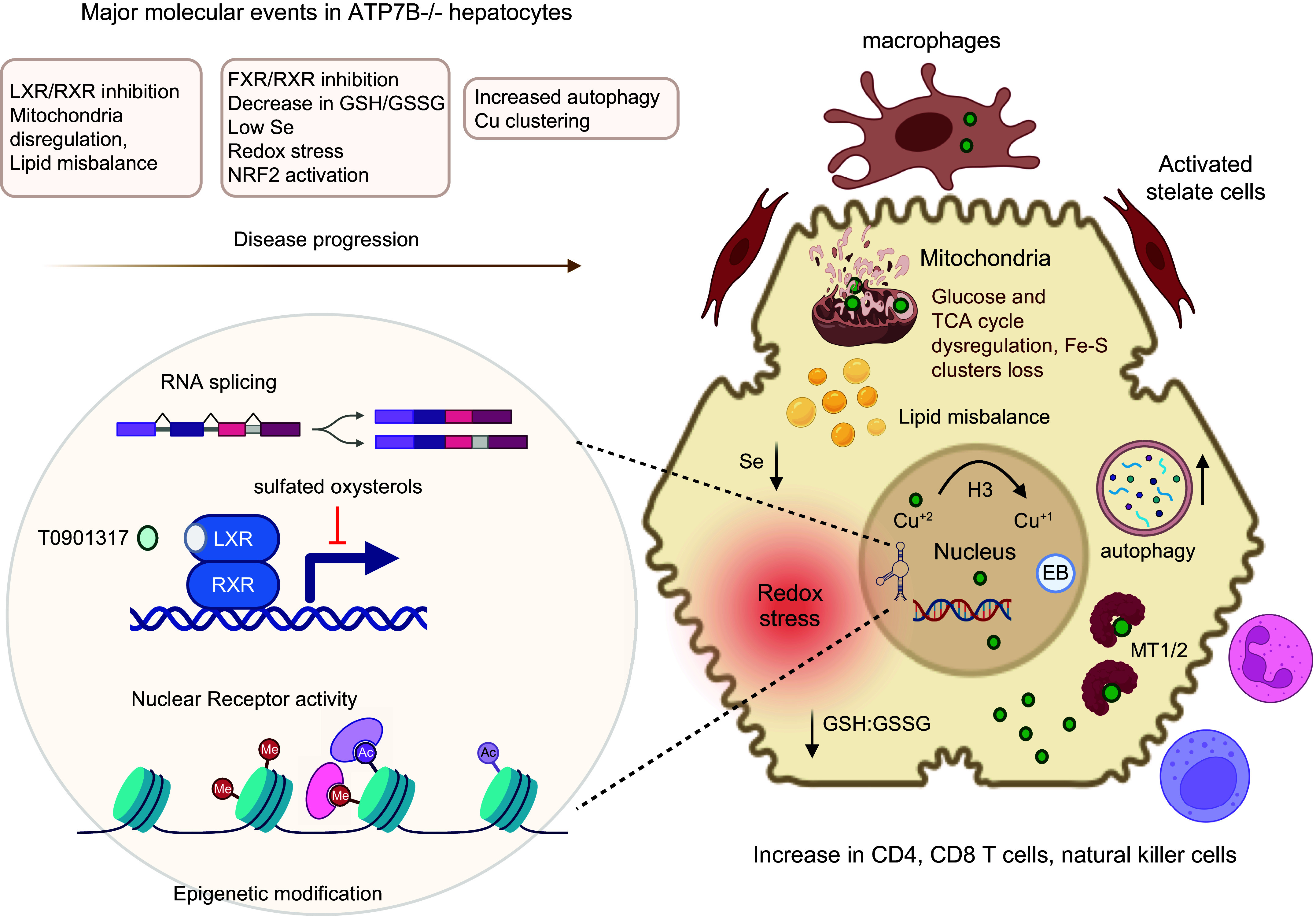
Molecular events in ATP7B^-/-^ hepatocytes leading to Wilson’s disease pathology. In the absence of ATP7B function, copper (Cu) accumulates in the cytosol (where it is sequestered by metallothioneins MT1/MT2), in mitochondria (where it inhibits mytochondria function), and in the nucleus causing changes in RNA splicing and LXR/RXR activity. Later, when Cu-binding capacity of MT1/2 is saturated, GSH:GSSG ratio decreases, leading to increased oxidated stress further augmented by low selenium (Se) levels. This causes upregulation of NRF2, further decrease in nuclear receptor activity (FXR/RXR) and mitochondria deterioration, and then relocation of transcription factor EB in the nucleus and activation of autophagocytosis. Image created with BioRender.com, with permission.

The hepatic aspect of Wilson’s disease has been relatively well characterized, and a number of excellent reviews describe disease pathogenesis and treatment in detail (115, 410–412). ATP7B is the major Cu efflux system in the liver. Inactivation of ATP7B results in time-dependent Cu accumulation in tissue, diminished mitochondria function, and lipid misbalance caused by inhibition of nuclear receptors. These changes in turn lead, eventually, to an increased inflammatory response, fibrosis, and, if untreated, liver failure (162, 412, 413) (FIGURE 14).

Studies in the animal and cell models of WD also highlighted the significance of ATP7B function in the intestine. Inactivation of Atp7b in the mouse intestine alters the processing of dietary fat and modifies liver metabolome, thus contributing to Wilson’s disease pathogenesis (180, 414). In fact, animals with Atp7b inactivated only in hepatocytes have much milder phenotypes than Atp7b global knockouts on the same genetic background, despite similar Cu accumulation in the liver (415). Future single-cell sequencing studies promise to provide additional details on the contribution of extrahepatic cells and tissues to WD.

Neurological and psychiatric WD is the least understood and is harder to diagnose and treat (115, 416–419). A combination of symptoms, including tremors, dystonia, parkinsonism, ataxia, and dysarthria are common for these patients (420). Cu chelation therapy as well as Zn replacement, which often provide significant benefit to patients with hepatic manifestations, can be less helpful for neurologic patients and even trigger neurologic deterioration. A new, methanobactin-based chelator, ARBM101, has been shown to quickly remove excess copper from the liver of the rat WD model via a biliary excretion (413). Liver transplantation was shown to benefit some chelation-resistant WD patients with severe neurologic deterioration (421), and further systematic studies are needed to better evaluate this treatment (416). Similarly, gene therapy using a miniaturized version of ATP7B has shown promising results in rodents (421–423). Phase I/II clinical trials are currently underway to assess the safety and pharmacological activity of the adeno-associated virus (AAV) vector-based gene therapy using miniaturized ATP7B in adults with WD. These important developments highlight the urgent need to better understand how tissues communicate their Cu status and how liver Cu homeostasis impacts other tissues. In addition, the potential immune response against the vector, decrease of protein expression over time, and relatively low efficiency of gene delivery have prompted the search for additional modalities (for review, see Ref. 424).

### 5.3. Cu Imbalance Is Reported in an Increasing Number of Brain Disorders

Besides Menkes disease and Wilson’s disease (238, 394), Cu misbalance has been reported in several other neurodegenerative disorders. Altered Cu homeostasis is observed in Alzheimer’s disease (AD), Parkinson’s disease (PD), Huntington’s disease (HD), amyotrophic lateral sclerosis (ALS), and spongiform encephalopathies (425). The origin and specific contribution of Cu misbalance in most of these disorders are subjects of significant interest and a growing number of studies. In Parkinson’s disease (PD), Cu deficit was repeatedly observed in the substantia nigra (426, 427) and especially in neuromelanin-positive neurons (428). Neuromelanin is a product of the nonenzymatic oxidation of dopamine and is more abundant in PD patients (429). Curiously, forced overexpression of Cu-dependent tyrosinase (an enzyme involved in the synthesis of neuromelanin) is associated with PD-like symptoms, including hypokinesia, formation of Lewy body-like, and nigrostriatal neurodegeneration (327). Tyrosine is a common precursor for the biosynthetic pathways that lead to the generation of dopamine (via tyrosine hydroxylase) and neuromelanin (via tyrosinase). Both pathways require Cu (by DBH and tyrosinase, respectively). Lower activity of dopamine-β-hydroxylase due to Cu deficiency or decreased expression of this enzyme increases the relative abundance of dopamine to downstream metabolites. Also, low levels of ceruloplasmin (a common indicator of Cu deficiency) were shown to correlate with a younger age of PD onset (430).

AD is a progressive neurodegenerative disease associated with the aggregation of β-amyloid plaques (Aβ40 and Aβ42) in the brain, especially in the cortical areas, and with tau-containing intracellular neurofibrillary tangles (431). The most common presentation is amnestic cognitive impairment. A significant amount of literature reported metal disturbances in AD (432–437). The amyloid precursor proteins APP and APLP2 were shown to have Cu and zinc binding sites (438, 439). This property can be used for diagnostic imaging of amyloid plagues, as the plaques entrap metals (440–442). The presence of metallic copper, which has distinct chemical properties, was recently discovered in AD brains (442). However, in the animal model of AD, neither Cu chelation nor elevation of Cu via diet had a significant impact on tau pathology (443), and the unifying mechanism of Cu contribution to AD pathogenesis has not yet emerged. Further studies are needed to determine whether Cu misbalance is a significant factor in AD pathogenesis or one of many contributing and modifying factors.

Amyotrophic lateral sclerosis (ALS) is a neurodegenerative disease of motor neurons with complex etiology. In addition to familial ALS associated with mutations in SOD1, other Cu-related phenomena were reported. Increased levels of Cu were found in the CSF of ALS patients and in the spinal cord of ALS mice (444–446). The expression of Atp7a and metallothioneins is downregulated in the spinal cord of ALS mice suggesting dysregulated Cu homeostasis. Recently, a patient with a slow-progressing ALS condition was identified with a novel mutation in the P-domain of ATP7A (447). Comprehensive analysis of the Cu handling machinery in ALS neurons may help to get a clear picture of Cu’s contribution to this disorder.

Huntington’s disease is a progressive neurodegenerative condition caused by poly-glutamine(Q) repeat expansion of Huntingtin (HTT) protein (448). The NH_2_-terminal of HTT binds Cu, and increased Cu levels promote aggregate formation of mutant HTT (449). Although the significance of this phenomenon in HD pathogenesis is still unclear, it is worth noting that the postmortem brains of HD patients and in experimental animal models show Cu accumulation in the striatum (450).

Prion (PrPc) is a glycosylphosphatidylinositol (GPI) anchored plasma membrane protein highly expressed in the central nervous system. Genetic mutations in the PRNP gene in humans cause neurodegenerative Creuzfeldt-Jakob disease. Prion is also associated with transmissible spongiform encephalopathies. Altered levels of Cu were observed in the prion-infected brain tissues. PrPC binds Cu with high affinity; Cu stabilizes prion protein by promoting β-sheet structure, enhances protease resistance, and triggers endocytosis (451). However, the role of PrPc in Cu metabolism remains unclear.

## 6. THE ROLE OF COPPER IN CELL PROLIFERATION, DIFFERENTIATION, AND REGULATED CELL DEATH

### 6.1. The Role of Copper in Cell Differentiation

The Cu deficit and excess have significant effects on cell proliferation and functional maturation, both in vitro and in vivo (62, 321, 452–455). These effects reflect the requirement for Cu during specific steps in cell functional development. During myogenesis, Cu is required for both proliferation and differentiation (108), and Cu uptake via CTR1 is upregulated (160). Within cells, Cu distribution to mitochondria and to the secretory pathway is enhanced via upregulated SLC25A3/Pic2 (252) and ATP7A, respectively. ATP7A supplies Cu to lysyl oxidase, which is required for myotubes formation (160). Similar upregulation of CTR1 and ATP7A is observed in differentiating adipocytes (53), neuronal cells (106, 356), and primary spermatocytes (456, 457). Higher expression of CTR1 and ATP7A increases Cu flow to the secretory pathway, which is necessary to accommodate strong upregulation of Cu-dependent enzymes (LOX, DBH, PAM, and others), which are necessary for specific cell functions (see above). In addition, signals inducing cell differentiation can trigger temporary trafficking of ATP7A from the *trans*-Golgi network to vesicles, which in this case can be Cu independent and serve to temporarily decrease the cytosolic Cu content. In neuronal cells, such trafficking takes place days before the upregulation of Cu-dependent enzymes and appears to be required for the maintenance of the reductive environment (106). The ATP7A interactome studied in SH-SY5Y cells has identified proteins involved in the formation of the growth cone, neuronal projection, and dendrite formation (29, 326, 347, 458, 459). In adipocytes, ATP7A function is required for termination of b-catenin signaling, which is inhibitory to differentiation (158).

While expression of ATP7A and CTR1 is often coregulated to maintain directional Cu flow inside the cell, ATP7B’s role during differentiation seems to generate and maintain Cu reservoirs. This reservoir is used for cellular activities that do not require Cu-dependent enzymes, such as chylomicron formation in enterocytes (180). In enterocytes, Cu depletion or the loss of ATP7B does not impact cell differentiation but markedly alters the processing of dietary fat (180, 344). Curiously, excess fat generates a condition of Cu deficit (460), demonstrating one of many links between copper and lipid metabolism (for reviews, see Refs. 461, 462).

### 6.2. The Role of Cu in Cell Death

Since Cu is both essential and toxic at high enough concentrations (463–465), it is therefore not surprising that both copper overload and deficiency can result in cell death (463, 466). Over the years, Cu and Cu ionophores (small molecules that are used to deliver Cu into cells) were shown to induce cell death in bacteria (467, 468), yeast (469, 470), and cancer cell lines (471–473) by diverse mechanisms that were sometimes contradictory. These mechanisms include the induction of apoptosis (471, 474–476), nonapoptotic cell death including caspase-independent cell death (477–481), ROS induction (472, 482, 483), destabilization of Fe-S cluster proteins (484–487), and inhibition of the ubiquitin-proteasome system (474, 488–491). This multitude of diverse pathways may reflect either a true variety of physiological responses or, instead, be the off-target effects of a nonphysiologically high copper used in some of these studies. Recently, the term cuproptosis was coined to describe a specific, new form of regulated cell death that is induced by ionophores that bring copper to the mitochondria. This regulated mechanism of cell death can be induced at very low concentrations (nM range) of copper ionophores complexes and involves the dual targeting of lipoylated and Fe-S cluster-containing proteins in the mitochondria (discussed below). Currently, cuproptosis refers to the mitochondria-centered copper-dependent mechanism of killing human cells. However, the role of copper in promoting cell death could be much greater, involving additional yet underappreciated mechanisms that can be delineated into the following subcategories: *1*) copper ionophore-induced cell death (cuproptosis); *2*) genetically induced death (as a result of mutations in genes regulating copper importer/export and chaperoning; and *3*) metabolism-dependent copper-mediated cell death (dysregulation of metabolic “chaperones” of copper, such as glutathione). These are discussed below.

### 6.3. Cuproptosis

Cuproptosis is a term that has been recently coined to describe a unique form of regulated cell death. Cuproptosis is induced by specific ionophores that cause accumulation of copper in the mitochondria while bypassing the copper uptake transporter SLC31A1 (226) and the mitochondrial Cu importer SLC25A3 (251, 492) Using pulse treatment of cells with low doses of such ionophores, it was first established that even a transient exposure results in an irreversible commitment to cell death at later time points (24–72 hours), i.e., the death is regulated. Second, this copper-dependent cell death could not be blocked by genetic or chemical inhibition of known cell death pathways such as apoptosis (BAK/BAX and caspase inhibition), necroptosis, (RIPK signaling), and ferroptosis (lipid antioxidants) or any form of caspase-mediated cell death (Pan-caspase inhibitors) (473). The whole genome CRISPR/Cas9 gene deletion screens revealed that the key regulators of cuproptosis are FDX1 (a mitochondrial ferredoxin), the lipoylating enzymes (LIPT1, LIAS), and the downstream lipoylated protein complex (the pyruvate dehydrogenase complex) (473, 477). This is consistent with the regulation of cuproptosis by cellular metabolism. Forcing cells to a state with increased mitochondrial metabolism by reducing the levels of glucose in the media increases the sensitivity to cuproptosis by orders of magnitude possibly due to increased reliance on the TCA cycle metabolism and protein lipoylation. Conversely, inhibition of electron transfer chain, pyruvate uptake to mitochondria or reduction in oxygen availability all effectively attenuated the induction of cuproptosis.

#### 6.3.1. FDX1-dependent cuproptosis.

To date, FDX1 is the best-characterized regulator of cuproptosis. Gene deletion of FDX1 confers resistance, whereas increased gene expression of FDX1 across 724 cancer cell lines is the top correlate with increased sensitivity to treatment with elesclomol, a mitochondria-targeted copper ionophore (477). Two main functions of FDX1 contribute to its ability to regulate cuproptosis. The first is that FDX1 directly reduces Cu(II) bound to ionophores to Cu(I), resulting in the release of Cu and possibly the recycling of the ionophore (477, 493). Second, FDX1 is a key upstream regulator of protein lipoylation (494–496): it enables the radicalization of SAM, which is required to initiate the enzymatic lipoylation reaction by the lipoylation enzyme LIAS. The role of FDX1 as an essential regulator of protein lipoylation in the cell may explain its role as the predominant cuproptosis regulator. Downstream of FDX1, copper directly binds and induces the aggregation of lipoylated proteins and promotes the global destabilization of Fe-S cluster proteins (473) consistent with some previous findings in bacteria (484–487). The genetic data indicate that copper-mediated cell death in cells does not occur due to the inhibition of protein lipoylation but rather that an active cellular lipoylation pathway is required to promote copper-induced toxicity and cell death. In other words, if cuproptosis occurred solely due to inhibition of cellular lipoylation (as suggested by in vitro results; Ref. 494), deletion of lipoylation regulating enzymes would phenocopy the function of the copper ionophores and promote cell death. This is not the case, and the opposite is observed; the lack of active FDX1 and lipoylation pathway enzymes rescues from cuproptosis (473). This suggests a model whereby FDX1-regulated lipoylation enables a toxic-gain-of-function mechanism of copper, which drives cuproptosis. These mechanisms are still not fully characterized and could involve either cell damage induced by lipoylated protein aggregates, toxicity arising from Fe-S cluster breakdown (including that of the lipoylating enzyme LIAS), or the formation of a unique copper-mediated toxic protein or metabolite intermediates (as discussed in Refs. 497, 498). Establishing these mechanisms is the next crucial step in the mechanistic definition of cuproptosis.

**FIGURE 15.**
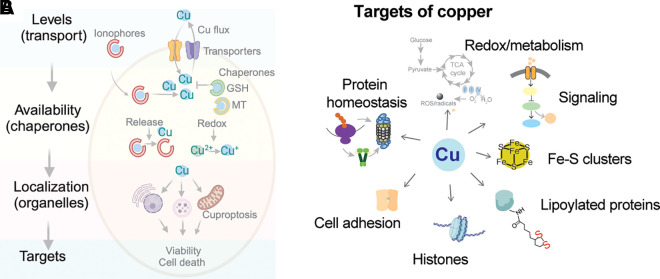
Mechanisms driving copper (Cu)-regulated cell death. *A*: levels of Cu are dictated by the natural Cu flux regulated by specific Cu importers/exporters and the efficiency and abundance of specific Cu ionophores. Availability and reactivity of Cu are regulated by mechanisms of Cu release from its protein/metabolite/ionophore bound state and its redox regulation dictating its reactivity and binding partners. Intracellular localization of Cu in specific organelles will dictate the downstream targets of Cu and the resulting mechanisms of toxicity. *B*: targets of Cu in the cell as previously characterized include the targeting of signaling cascades (such as Cu-binding kinases); mitochondrial Cu accumulation will result in targeting of lipoylated and Fe-S cluster-containing proteins. Cu directly affects protein homeostasis by directly engaging with the ubiquitin-proteasome system machinery and/or by facilitating the aggregation of proteins. Cu can also directly target metabolism and redox-regulating enzymes promoting metabolic and redox stress, cell adhesion, and histone biology. Image created with BioRender.com, with permission.

For an ionophore to be a selective inducer of cuproptosis it needs to have the following traits. First, it requires strong selective binding to copper. Elesclomol and disulfiram (DSF) (499) both bind copper, with elesclomol exhibiting particularly high affinity (*K*_a_ = 10^17.1^) at physiological pH (500). Second, the ionophore needs to possess the physical properties that promote copper delivery to the mitochondria. This property has been demonstrated for both elesclomol and to a lesser extent disulfiram using chemical screens in yeast systems, where selective copper shuttling to mitochondria boosts respiratory growth (501) and rescues genetic mitochondrial copper deficiency (58, 501). Finally, based on our current mechanistic understanding of cuproptosis, the copper bound to the ionophores needs to be reduced and released, a process that at least in part is regulated by FDX1 (477, 493). However, it is plausible that not all mitochondria targeting ionophores are reduced efficiently by FDX1, like elesclomol, as recently demonstrated (493). As such, for other copper ionophores [ATSM (502), GTSM (503), NSC-319726 (472), 8-HQ (479), Pyrithione (504), and more (505, 506)], there may exist additional mechanisms of reduction and release that are still unknown. Upon release of copper from the ionophore, the site of release will largely dictate the toxicity that will arise. In yeast and bacteria, copper-mediated cell death was attributed to the targeting (484–486) and inhibition of biosynthesis (486, 507, 508) of Fe-S clusters. In human cells, copper released from ionophores targeted to mitochondria preferentially targeted lipoylated and Fe-S cluster proteins (473). Copper released in other organelles or cytoplasm will be expected to target additional pathways (FIGURE 15).

#### 6.3.2. Other forms of cuproptosis.

The current definition of cuproptosis largely refers to the specific mechanisms that involve copper ionophores that target copper to the mitochondria (473, 477). Other copper homeostasis dysregulation states that result in copper-dependent cell death may involve additional regulatory mechanisms. These cell states may include the genetic induction of cuproptosis by dysregulation of cellular copper flux, which is normally regulated by the copper importer SLC31A1 (226) and the copper exporters ATP7B and ATP7A (described in other sections of this review). The elevation of intracellular labile (and reactive) copper pools due to a reduction in either protein copper chaperones such as ATOX1 and metallothionines (24, 509, 510) or the metabolite chaperone of copper such as glutathione (269, 473, 511). Beyond the transcriptional or metabolic changes that may increase the labile, toxic pool of intracellular copper, the intracellular (or organelle) redox state may also be a regulatory mechanism of copper-induced toxicities and cell death (512–515). Elucidating the broad spectrum of these different copper-regulated cellular processes and corresponding cytotoxicities will further contribute to the broadening of the mechanistic definition of cuproptosis.

### 6.4. Copper in Cancer

For many decades, the unique dependency of highly proliferating cancer cells on specific essential nutrients has been exploited to develop antimetabolite drugs that have been very successful in the clinic (516). Copper is both an essential element for life (463) and a highly reactive and toxic element at high concentrations. As such, copper homeostasis follows the Goldilocks principle, where both too much and too little copper can result in a lack of proliferation and death of cancer cells. This principle has been used to explore distinct anticancer therapeutic modalities involving copper homeostasis. The first approach is based on the antimetabolite principle: copper chelators are used to induce copper deficiency in cancers that are particularly dependent on high copper levels. The second modality exploits copper ionophores to induce copper overload that may synergize with specific cancer cell metabolic states to promote cancer cell death.

#### 6.4.1. Copper chelation as an anticancer therapeutic strategy.

This strategy stems from the concept of antimetabolite drugs, i.e., since copper is an essential element, chelation of copper will result in cancer cell death. Supporting this strategy, there has been increasing evidence that patients with specific cancers have either elevated copper in serum and, in some cases, tumors (517–525), or, as reviewed in Refs. 464, 465, specific cancers may depend on higher copper availability to promote their proliferation. Copper chelators are well tolerated in healthy people and have been successfully used for the treatment of copper accumulation disorders such as Wilson’s disease (115). It is therefore tempting to try repurposing these chelators for the treatment of cancer (526, 527).

Over the years, several cellular mechanisms were proposed to explain the anti-cancer effects of copper chelators. Copper chelation was shown to inhibit mitochondrial metabolism by targeting the copper-dependent complex IV, resulting in reduced cancer cell proliferation in cell culture and in xenograft models in vivo (152, 528, 529). Copper chelators were also shown to inhibit the metastatic potential of cancer cells by limiting Cu delivery to LOX enzymes that are essential for the remodeling of the extracellular matrix (273, 303, 530–532) and by targeting angiogenesis through mechanism involving VEGF (533–537). More recently, copper was shown to positively regulate several kinases that contribute to oncogenesis (465). These include the oncogenic kinase MEK in the RAF-MEK-ERK signaling cascade (128, 538–542) and ULK1/2 kinases regulating autophagy (125, 542). Copper also directly regulates cellular immune response by increasing levels of PD-L1 and through other mechanisms (531, 543). These discoveries of diverse copper-dependent enzymes and signaling pathways that promote oncogenesis set the foundation for the initiation of numerous clinical trials to test whether a copper chelation strategy could be a useful anticancer therapeutic modality.

To date, most clinical trials using copper chelators did not achieve the desired results (544–547), despite being well tolerated and achieving desired systemic copper levels reduction. One potential explanation for this lack of efficacy is the prolonged treatment time necessary to achieve copper depletion and tumor growth inhibition (527). Additionally, many cancers exhibit a rewiring of cellular metabolism, showing high levels of glycolysis even in the presence of oxygen, a phenomenon known as “aerobic glycolysis” (Warburg effect) (548). However, this does not mean that cancers are not dependent on functional mitochondrial metabolism and respiration (549, 550). As copper chelation reduces levels of mitochondrial complex IV, understanding the altered dependency of cancers on mitochondrial respiration is crucial for the success of this therapeutic approach (and that of the copper ionophores described below). Despite these considerations, the copper chelation approach showed success in a more recent phase II clinical trial of breast cancer patients who were at high risk of recurrence and were treated with TTM following initial chemotherapy (530). Particularly promising results in this trial were achieved for triple-negative breast cancer (TNBC) patients. This lethal cancer subtype is responsible for ∼50% of the metastatic tumors in breast cancer patients and has an overall average 5-yr survival of 11%. Copper chelation produced a 2-yr event-free survival (EFS) for stage II/III TNBC of 90% and stage IV with no evidence of detectable disease (NED) of 69% (527, 530). These promising results facilitated the currently ongoing clinical trials focused on TNBC patients.

#### 6.4.2. Targeted copper delivery as a therapeutic strategy.

Copper overload results in cell death and as such was proposed as a therapeutic strategy to target specific cancers (473, 477, 551, 552). Elesclomol and disulfiram are potent copper ionophores that are safe for use in humans, as demonstrated in clinical trials for elesclomol (553–556), or as an approved therapy as in the case of disulfiram (557). This motivated the initiation of multiple clinical trials over the years, aimed at testing the efficacy of these compounds as a potential anticancer therapeutic. However, the design of these trials was strongly undermined by the lack of understanding of the mechanism of action of these compounds.

##### 6.4.2.1. elesclomol as an anticancer agent.

Elesclomol and a sodium salt formulation of elesclomol have been tested in several clinical trials of patients with advanced melanoma, ovarian cancer, and acute myeloid leukemia (553–556, 558) and other unpublished trials (clinicatrial.gov). The initial small, randomized phase II study where elesclomol was given to patients with metastatic melanoma in combination with paclitaxel (Taxol) showed significantly increased progression-free survival (554). However, all other clinical trials resulted in the lack of desired clinical response (553, 555, 556, 558). Despite the failure in the phase III clinical trial, a post hoc analysis revealed that patients with low plasma lactate dehydrogenase (LDH) levels showed evidence of antitumor activity (553). Low LDH reflects higher cellular dependency on mitochondrial metabolism (as opposed to glycolysis), consistent with the recent mechanistic characterization of the mitochondrial pathways involved in cuproptosis. At the time of the clinical trial design and execution, it was thought that the major anticancer mechanism of elesclomol was the elevation of intracellular reactive oxygen species targeting multiple downstream pathways (482, 551, 559–567) and strong induction of the heat-shock response (475, 564, 568, 569). Only later was it established that elesclomol bound copper and served as a potent copper ionophore (482, 570) specifically targeting copper to the mitochondria (58, 482), wherein an FDX1-dependent manner (477, 493), it promotes copper-dependent cell death by targeting lipoylated and Fe-S cluster proteins (473, 477, 494). Thus the current mechanistic understanding of elesclomol-induced cell death will enable mechanism-focused criteria for patient selection in any future clinical trial design.

##### 6.4.2.2. disulfiram as an anticancer agent.

Disulfiram [tetraethylthiuram disulfide (DSF); Antabuse] is another copper ionophore that has been developed and approved for use in the United States for more than seven decades to treat alcohol dependence, with well-established pharmacokinetics, and FDA-approved safety profile (557). Disulfiram is a member of the dithiocarbamate family that contains sulfur-based chelators that have been described to induce cell death by mechanisms involving increases in oxidative stress, which is dependent on copper uptake (471, 476, 571). Over the years the anticancer properties of disulfiram were described in cell culture (572, 573), and a retrospective observational study of over 240,000 cases from 2000–2013 found that cancer-related mortality, for all cancers, was significantly reduced for continuing users of disulfiram compared to patients who previously used disulfiram (488). As in the case of elesclomol, the known mechanism of disulfiram-induced cytotoxicity was diffuse, preventing a mechanism-based approach resulting in trials that did not achieve desired endpoints (552, 574–576), whereas one small trial resulted in a hint of efficacy when disulfiram was used in combination with chemotherapy (577). Disulfiram was shown to directly target and inhibit acetaldehyde dehydrogenase (578), the ubiquitin proteins system (579), and caspases (580, 581) and more recently was shown to induce cuproptosis by targeting lipoylated and Fe-S cluster proteins. Analysis of cell killing prolife of disulfiram across hundreds of cancer cells showed that both copy number loss of chromosomal arm 16q and low expression of the metallothionein-encoding genes MT1E and MT2A residing in this genomic region, correlate with disulfiram-induced cell killing (573). However, disulfiram is a drug that is highly metabolized, making its clinical utilization for cancer therapy even more challenging (582).

The increasing appreciation for the regulatory role of copper in cancer progression and the specific cell death mechanisms that can be evoked by copper overload may both serve as orthogonal approaches to harness copper biology to target specific cancers. The success of any future trial will depend on achieving the desired pharmacokinetics properties of the molecules, mechanism-based patient stratification strategies, and combination with therapeutics that promote synthetic lethality.

## 7. CONCLUSIONS

The field of copper biology is rapidly expanding revealing physiological and clinical importance as well as the complexity of human copper physiology. Future studies will shed light on molecular mechanisms behind copper-mediated signaling and will identify the nature of copper storage compartments and small copper carriers in biological fluids. We will learn more about the metabolic connections of copper and the role of copper in cell motility and metastasis. A better understanding of copper biology, especially in the central nervous system, will open additional avenues for diagnostics and treatment of disorders, in which copper dys-homeostasis is a contributing factor. It is clear that future studies will bring many new and exciting discoveries.

## GRANTS

This work was supported by the National Institutes of Health Grants R01DK117396 (to S.L.), R01DK071865 (to S.L.), and R01NS134958 (to S.L.), National Cancer Institute Grant R01CA279550 (to P.T.), and Office of Naval Research (ONR) Grant N00014-23-1-2465 (to P.T.).

## DISCLOSURES

No conflicts of interest, financial or otherwise, are declared by the authors.

## AUTHOR CONTRIBUTIONS

S.R. and P.T. prepared figures; S.L., S.R., and P.T. drafted manuscript; S.L., S.R., and P.T. edited and revised manuscript; S.L., S.R., and P.T. approved final version of manuscript.
